# Pioneer statoacoustic neurons guide neuroblast behaviour during otic ganglion assembly

**DOI:** 10.1242/dev.201824

**Published:** 2023-11-08

**Authors:** Aitor Bañón, Berta Alsina

**Affiliations:** Department of Medicine and Life Sciences, Universitat Pompeu Fabra-Parc de Recerca Biomèdica de Barcelona, Dr Aiguader 88, 08003 Barcelona, Spain

**Keywords:** CRISPR, Statoacoustic ganglion, Zebrafish, Inner ear, Delamination, Migration, RhoGTPases, Neuroblasts

## Abstract

Cranial ganglia are aggregates of sensory neurons that mediate distinct types of sensation. The statoacoustic ganglion (SAG) develops into several lobes that are spatially arranged to connect appropriately with hair cells of the inner ear. To investigate the cellular behaviours involved in the 3D organization of the SAG, we use high-resolution confocal imaging of single-cell, labelled zebrafish neuroblasts (NBs), photoconversion, photoablation, and genetic perturbations. We show that otic NBs delaminate out of the otic epithelium in an epithelial-mesenchymal transition-like manner, rearranging apical polarity and primary cilia proteins. We also show that, once delaminated, NBs require RhoGTPases in order to perform active migration. Furthermore, tracking of recently delaminated NBs revealed their directed migration and coalescence around a small population of pioneer SAG neurons. These pioneer SAG neurons, not from otic placode origin, populate the coalescence region before otic neurogenesis begins and their ablation disrupts delaminated NB migratory pathways, consequentially affecting SAG shape. Altogether, this work shows for the first time the role of pioneer SAG neurons in orchestrating SAG development.

## Teaser

**Highlighted Article:** High-resolution confocal imaging and migration tracking of zebrafish neuroblasts reveals the cellular behaviours underlying statoacoustic morphogenesis and the role of pioneer neurons shaping the statoacoustic ganglion.

## INTRODUCTION

The inner ear is responsible for the senses of hearing and balance and is organized into two main structures: the epithelial labyrinth containing the hair cells and the statoacoustic ganglion (SAG). During development, cranial sensory ganglia undergo cellular rearrangements in a process of morphogenesis to acquire their final functional shape. The SAG develops into a particular architecture with three distinct lobes that establish topographic projections with the sensory patches of the inner ear: two anterior lobes elongated dorsoventrally, and a posterior lobe elongated anteroposteriorly (Taberner et al., 2018). For correct circuitry between hair cells and otic neurons, SAG morphogenesis must be tightly coordinated in time and space with otic tissue development (Bok et al., 2013).

Several studies, using zebrafish as a model organism, have addressed the cell shape dynamics underlying the 3D sculpturing of the inner ear labyrinth. By light sheet microscopy, it has been shown that semicircular canal formation depends on extracellular matrix expansion and filopodia (Jones et al., 2022), lumen expansion requires anisotropic epithelial thinning, and endolymphatic duct growth depends on lamellar projections (Hoijman et al., 2015; Swinburne et al., 2018). In contrast, morphogenesis of the SAG is less well understood. Live imaging of SAG neurons is a challenging task because neurons position behind the otic vesicle and they rapidly compact into a ganglion. For that reason, SAG neurons have mainly been treated as a ganglionic unit without a detailed analysis of individual SAG neuronal dynamics and shapes.

The inner ear primordium, the otic placode, consists of an epithelium with an internal cavity, or lumen. Otic progenitors display interkinetic nuclear migration and their apical membrane is oriented to the lumen, whereas the basal membrane aligns with the basal lamina (Alvarez et al., 1989). At the anteroventral quadrant of the otic vesicle, the neurogenic domain emerges with the competence to specify neuronal precursors (Abelló et al., 2010; Hoijman et al., 2017). Neurogenesis initiates anteroventrally, but progressively extends to posteromedial positions, as observed by the temporal pattern of expression of *neurogenin1* (*neurog1*) in zebrafish, mouse and chick (Abelló et al., 2007; Bok et al., 2007, 2011; Radosevic et al., 2011). Later, Neurog1^+^ cells transit into Neurod^+^ cells and begin their delamination out of the otic epithelium (Ma et al., 1998; Sommer et al., 1996). In chick and zebrafish, the most anterolateral portion of the neurogenic domain generates vestibular neurons, whereas the posteromedial region gives rise to auditory neurons (Bell et al., 2008; Dyballa et al., 2017). Whether or not otic neuroblast (NB) delamination follows an epithelial-mesenchymal transition (EMT) programme has been under debate. Some authors suggest that core EMT transcription factors and RhoGTPases (specifically RhoB) are not used in placodal sensory neuron delamination (Graham et al., 2007). Additionally, the migratory capacity of cranial placode NBs is reduced relative to neural crest cells (Schlosser, 2008). However, EMT genes such as *snai1b*, *cadherin 6* and *Sox9/10* family genes are expressed in delaminating Neurod^+^ otic NBs and/or otic domains in zebrafish (Chiang et al., 2001; Dutton et al., 2009; Piloto and Schilling, 2010), suggesting otic NBs might be undergoing EMT.

Scattered single-cell labelling of membranes, nuclei and/or specific cellular components has revealed information on cell shape changes and behaviours during cell delamination or migration in the developing central nervous system (Hadjivasiliou et al., 2019; Kasioulis et al., 2022; Moore et al., 2013). One such behaviour is the modulation of neuronal dynamics by pioneer cells. These pioneer cells, first described in invertebrates, are defined as early-born cells that have a scaffolding capacity to organize the tissue and pre-form the final configuration (Aigouy et al., 2008; Karkali et al., 2023; Wanner and Prince, 2013; Whitlock and Westerfield, 1998). However, whether other cells also participate in SAG formation is unknown.

Here, we address SAG morphogenesis by combining single-cell labelling, photoconversion, photoablation, and genetic perturbations. Our data suggest that, indeed, otic NB delamination is an EMT event. We track and analyse cell shape changes in individual NBs during their migration before coalescence and reveal that their migration is an active mechanism. We also find that otic NB migration depends on RhoGTPases. Interestingly, NBs migrate and coalesce in a precise region populated by SAG pioneer neurons that arrive before neurogenic delamination in the otic placode begins. These pioneer SAG neurons recruit otic NBs and are required for the organization of the SAG shape and size. Overall, the study provides novel data on how the SAG acquires its 3D organization and the underlying complex cellular behaviours of NB responsible for SAG development.

## RESULTS

### Live imaging of otic neuroblasts during delamination reveal complex and dynamic cellular behaviours

**Fig. 1.**
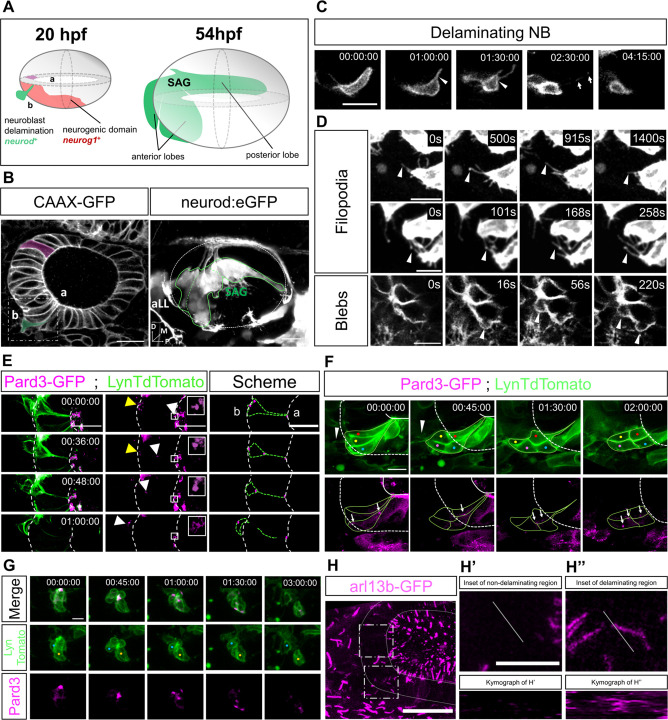
**Single cell labelling and imaging of delaminating otic NBs.** (A) Schematics of otic NB delamination in the neurogenic domain (red) at 20 hpf and SAG shape (green) at 54 hpf. (B) Labelling of the otic epithelial membranes at 24 hpf (left) and the SAG at 54 hpf (right), the latter being located behind the otic vesicle (white dashed oval). Both images are lateral views. An NB (pseudocoloured green) delaminating from the neurogenic domain displays a distinct shape compared with non-delaminating otic cells (pseudocoloured purple). The boxed area represents the region from where images in C and D are taken. Different SAG lobes are outlined with a green line. (C) Single-labelled delaminating NB undergoing apical thinning (white arrowhead). The NB exits neuroepithelia leaving membrane traces inside neuroepithelia (white arrows). The images shown in C are from Movie 1, and are also depicted in Fig. S1C (top) with Pard3-GFP fluorescence. (D) NB from the neurogenic domain extending dynamic filopodia and blebs (white arrowheads) (representative of *n*=7). See also Movie 2. (E) Delaminating NB undergoing apical thinning and Pard3 relocation. Pard3 moves from apical to basal domains concomitantly with membrane zippering (white arrowheads) and accumulation of Pard3 in basal domains (yellow arrowhead). Right-hand panel represents a scheme of this phenomenon. (F) Collective delamination of NBs. NBs extend filopodia in the collective front while delaminating (white arrowheads) and Pard3 relocates (white arrows) without losing its punctate pattern (representative of *n*=7). (G) After delamination, a group of contacting NBs separate (blue and orange dots) concomitantly with Pard3 rearrangements (representative of *n*=3). (H) Arl13 staining in magenta. Non-neurogenic (upper dashed square) and neurogenic (lower dashed square) anterior domains are indicated, representing the region of insets in H′ and H″, respectively, from a different embryo. The Arl13b reporter is observed inside the neuroepithelia in the neurogenic domain, but is absent in the non-neurogenic domain. A spatial section across time (white transverse line corresponding to kymographs) shows the presence of Arl13 reporter in the neurogenic region only (representative of *n*=10). a, apical; aLL, anterior Lateral Line ganglion; b, basal. Anterior is to the left, posterior to the right in all images. Scale bars: 20 µm (B,H); 10 µm (C-G,H′,H″).

Zebrafish NBs delaminate out from the neurogenic domain [depicted in red in an otic vesicle of 20 hours post-fertilization (hpf) in Fig. 1A] and generate the trilobular SAG behind the otic vesicle, as visualized with the transgenic line *Tg(neurod:eGFP)* (Fig. 1A, green; Fig. 1B, right). In the ventral neurogenic domain, pre-delaminating NBs remodel and acquire a different shape than cells in the non-neurogenic domain, which are more columnar (compare green with purple pseudocoloured cells in Fig. 1B).

We fluorescently labelled the membranes in single and scattered NBs for better characterization of the cell shape changes that occur during NB delamination by injecting lynTdTomato mRNA into one of the cells in 32- to 64-cell-stage embryos. *In vivo* imaging from 24 to 36 hpf, the time window of highly active NB delamination, showed how a delaminating NB progressively acquires a triangular shape and concentrates the cytoplasm basally (Fig. 1C,E). During this cell shape rearrangement, the apical cellular domain becomes narrower in a zipper-like process, to finally become a thin, membranous filament (arrowheads in Fig. 1C). In the neural tube, it has been shown that the differentiating neurons detach from the lumen by a process of apical abscission, whereby the cell is apically constricted and detached from the luminal membrane (Das and Storey, 2014). We also observed that when the basal cell body is mostly outside the epithelium, but not before, the attachment to the apical membrane is broken, and the delaminating NB loses its apical contact, leaving behind some leftover membrane (Fig. 1C, white arrows; Fig. 1E; Movie 1). High temporal resolution imaging (Fig. 1D) showed that pre-delaminating NBs generate a high number of dynamic filopodia inside and outside the neuroepithelium as well as blebs at the basal cellular domain lining the basal lamina (Fig. 1D, arrowheads from top to bottom rows, respectively; Movie 2). The basal lamina is disrupted at the neurogenic domain (Alvarez et al., 1989). To ascertain whether the apicobasal polarity is lost or rearranged during the delamination process, we assessed the localization of apical determinants during delamination. For this, lynTdTomato mRNA was co-injected with the apical protein *pard3* mRNA at the 32- to 64-cell stage. When analysing the Pard3 signal location in individual NB, a small fraction of the Pard3 signal remained at the abscised membrane that was left at the luminal area (Fig. 1E, insets in central panels), but another fraction was detected regressing with the plasma membrane thinning edge (Fig. 1E, white arrowheads). In addition, accumulation of Pard3 was also transiently detected in the basal cytoplasmatic domain in early delamination (Fig. 1E, yellow arrowheads in central panels), in contraposition to Pard3 in non-delaminating regions, which always remains apical, suggesting a possible change in polarity (Movies 1 and 3; Fig.  S1). When labelling was less mosaic, several cells delaminating collectively were observed (Fig. 1F). Some NBs delaminated more dorsally (red, yellow and blue dots) and others more ventrally (magenta dot, absent in first frame). Delaminating NBs extended filopodia (Fig. 1F, white arrowheads) and dynamically relocated puncta of Pard3 (Fig. 1F, white arrows). As shown in Fig. 1C,E delamination of NBs spanned for a period of 1.5-2 h.

Once NBs are completely outside, instead of being elongated, they acquire a more mesenchymal and rounded shape. In a group of already delaminated NBs contacting each other (Fig. 1G), Pard3 expression was initially concentrated at cell–cell contacts but then redistributed when NBs separated (Fig. 1G, blue and orange dots indicate cell bodies that separate through time).

Hence, Pard3 relocation during both delamination and migration suggests that polarity is reorganized and could contribute to NB delamination and dispersion (Fig. 1G; Movie 4). Interestingly, actomyosin contraction observed with the reporter line *Tg(actb2:myl12.1-mCherry)* (magenta or Fire LUT in Fig. S2) was detected in Pard3 (Fig. S2, green) vicinities, suggesting a spatial relationship and a possible functional connection between polarity dynamics and NB contraction during delamination.

During delamination of neural tube differentiating neurons, atypical protein kinase C (aPKC) and the primary cilia are retained in the ventricle membrane when the cell suffers apical abscission (Das and Storey, 2014). In contrast, zebrafish retinal neuroblasts keep the primary cilia at the apical front of the retracting cell or either dismantle it only shortly before retraction during delamination (Lepanto et al., 2016). To study the localization of the primary cilia during otic NB delamination, we used the *Tg(actb2: arl13B-GFP)* line. In the non-neurogenic and neurogenic regions (upper and lower dashed squares, respectively, in Fig. 1H), Arl13b staining was oriented towards the lumen in the non-neurogenic region and absent inside the epithelium, but present inside the neurogenic domain epithelium. This suggests that delaminating NBs carry the primary cilia with them when delaminating, whereas non-delaminating cells keep Arl13b in the apical site (Fig. 1H′,H″; Movie 5). In a kymograph of the area (spatial section of these regions across time, indicated by white transverse lines in Fig. 1H′,H″), Arl13b fluorescence crossed the spatial section of delaminating NBs but not into the non-delaminating region, again suggesting that Arl13b travels inside the neuroepithelium only in the delaminating region (kymographs in Fig. 1H′,H″; *x-*axis corresponds to the spatial section, stacked across time, represented by the *y*-axis).

Injection of mRNA at the 32- to 64-cell stage resulted in a few embryos with individual cells labelled. To increase the number of single NBs labelled, we used what we have called CRISPR Eraser. In these experiments, a guide RNA against GFP was injected into embryos of the *Tg(neurod:eGFP)* reporter line, which labels NBs. Because Cas9 is highly efficient, most cells have the GFP gene disrupted by Cas9, and only a few NB retain cytoplasmic eGFP expression, either by escaping Cas9 targeting or because of efficient repair restoring eGFP genomic sequence (see Materials and Methods and Fig. S3). This creates an environment in which most Neurod*^+^* cells of the developing SAG are eGFP negative and the remaining eGFP^+^ NB can be seen with high contrast.

**Fig. 2.**
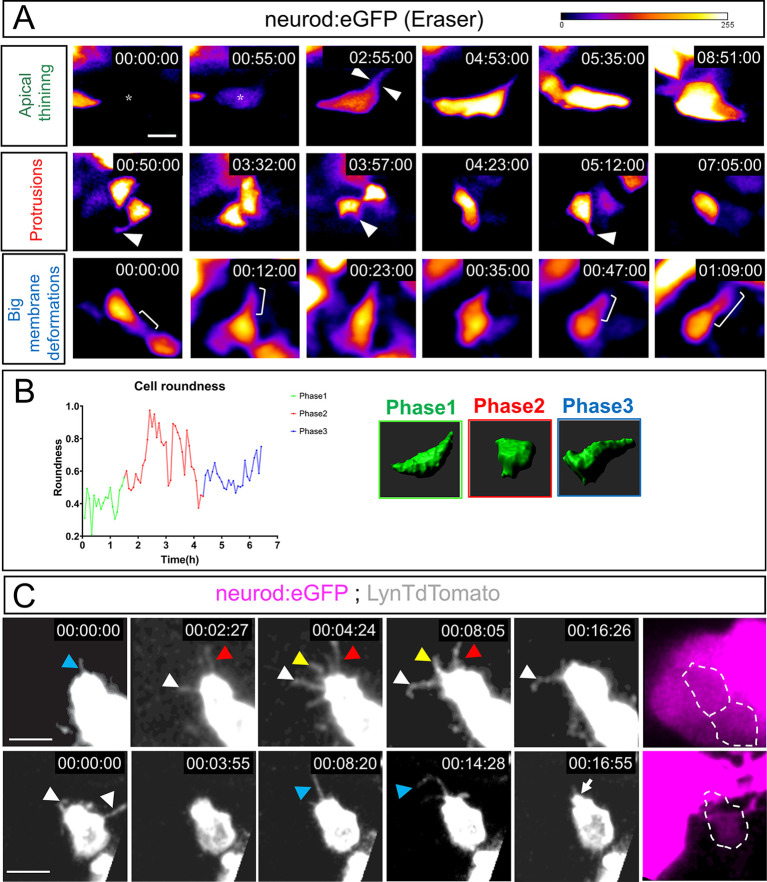
**Delaminated otic NBs dynamically change their shape, producing filopodia and membrane protrusions.** (A) Single-cell labelling of delaminating NBs with CRISPR Eraser in *Tg(neurod:eGFP)*. Delaminating NBs undergo apical thinning and delamination (white arrowheads in top row) right after starting to express *neurod* (asterisks). Subsequently, they acquire a much more rounded, mesenchymal shape and produce membrane protrusions (white arrowheads, middle row). Finally, membrane protrusions precede large membrane deformations (white brackets, bottom row). Representative of *n*=7. (B) Example of the change in roundness of one NB through the different phases. Phases are colour-coded and a 3D IMARIS reconstruction is shown on the right. (C) NBs produce dynamic filopodia after delamination (coloured arrowheads). Bottom row shows how a membrane protrusion becomes a much bigger membrane deformation (white arrow). Panels on the right show cells in left-hand panels depicted inside the dashed lines with eGFP staining from Neurod^+^ cells shown in magenta. Scale bars: 10 µm.

In CRISPR Eraser embryos, NB cell bodies change from an elongated and triangular shape to a rounded cell body by the time they exit the epithelium (Fig. 2A, white arrowheads in upper panels). Once delaminated, NBs modify their roundness, extend filopodial protrusions, produce large membrane deformations (Fig. 2A, white arrowheads in middle panels; Fig. 2C; Movie 6) and elongate (Fig. 2A, brackets in lower panels). During the whole timespan of the imaging of selected NBs, the roundness was quantified using Fiji and plotted in an example in Fig. 2B (see Fig. S4 for more examples). At least three different phases of cell shape variation can be distinguished: an initial phase corresponding to apical thinning and delamination; a second phase of increased roundness and membrane protrusions; and a final phase of elongation, presumably to engage into migration. Imaris 3D reconstruction of a paradigmatic example depicts these three stages (Fig. 2B).

In summary, delaminating NBs suffer a process of apical thinning and abscission, but Pard3 and Arl13b components are not lost and relocate in the delaminated cell, suggesting that this process can help establish a new polarity front in NBs. Moreover, NBs extensively deform their membranes with a large number of blebs, filopodia and larger protrusions, which might generate mechanical forces and/or establish cell communication events.

### Active migration of delaminated SAG neuroblasts is RhoGTPase dependent

Massive delamination of zebrafish otic NBs in the anterolateral region takes place between 17 and 30 hpf. Large groups of cells delaminate and position anterior to the otic epithelium (Hoijman et al., 2017). Thus, displacement of NBs within the SAG can be driven mainly by new delaminating NBs pushing on the previously delaminated cells. However, the dynamic changes in cellular shape and apicobasal polarity, together with the presence of filopodia observed in individually labelled NBs, suggests that delaminated NBs might display an active and directed migratory behaviour.

Most of the cellular deformations produced by cells to actively migrate involve the activation of RhoGTPases. In particular, the RhoGTPase Rac1 plays a role in directed migration and lamellipodia formation, Cdc42 in the acquisition of migratory capacity and filopodia formation, and Rho in stress fibres and rear contractility (Nobes and Hall, 1995; Reig et al., 2014; Shoval and Kalcheim, 2012; Yamao et al., 2015). To investigate the role of RhoGTPases in NB migration, we decided to manipulate this signalling pathway in NBs. To this aim, we generated a CRISPR knock-in Gal4 line in the endogenous locus of the *neurod1* (hereafter referred to as *neurod*) gene, which recapitulated the expression of the reporter transgenic line *Tg(neurod:eGFP)* (Fig. S5). We then overexpressed different dominant-negative (DN) and constitutively active (CA) forms of RhoGTPases in the new *Tg(neurod:Gal4)* transgenic line.

**Fig. 3.**
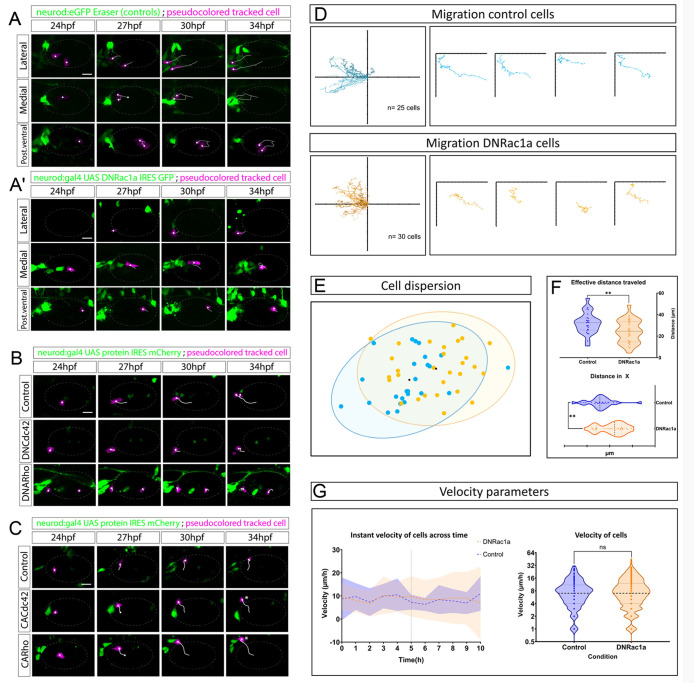
**Otic NBs engage in RhoGTPase-dependent active and directed migration.** (A,A′) Three NBs migrating from lateral, medial and posteroventral regions in control wild-type conditions under CRISPR Eraser in *Tg(neurod:eGFP)* (A) or in fish overexpressing DNRac1a (A′) (magenta pseudocoloured cells, tracks in white). See also Movie 7. (B) Migratory pattern under DNCdc42 and DNRho conditions. DNCdc42 and DNRho recapitulate the effect of reduced directed migration. (C) In contrast, CACdc42 and CARho show similar or even enhanced migration compared with control cells (asterisks). (D) Summary of tracked control and DNRac1a-expressing cells using DiPER. Tracks are normalized at the origin according to published protocols (Breau et al., 2017; Gorelik and Gautreau, 2014). Control cells migrate further and more directionally than DNRac1a cells. (E) Dispersion of cells (95% confidence interval) at the endpoint of the time lapse after normalization of tracks to the origin, in control (blue) and DNRac1a (orange) conditions. (F) Effective distance migrated is significantly compromised in DNRac1a compared with controls. This is measured as the length of a straight line between the start and end point of a given cell irrespective of their particular migratory path in the recordings. The distance covered in the *x*-axis is also compromised. Ticks in the *x*-axis represent increase every 10 µm. **P<0.005 (Mann-Whitney U-test for non-parametric data). (G) Instant velocities (space covered/time between frames) of delaminated NBs across time. The migratory capacity of cells is affected little because the mean (blue and orange dashed lines) of both control NB and DNRac1a NB is the same, although dispersion of standard deviation increases towards the end of the recording in the DNRac1a condition (orange area) versus controls (blue area). Black vertical dashed line indicates half of the recording time. For detailed tracked velocities, see Fig. S6. For DNRac1a analysis, the phenomenon was observed in *n*=25 control cells and *n*=30 DNRac1 cells, from 7 and 14 embryos, respectively. Scale bars: 20 µm. See Fig. S6 for individual cell migratory profiles. Regarding Cdc42 and Rho1 experiments, control cells of the DN condition migrate normally in 2/2 cases; DNCdc42 cells migrate normally in 4/7; DNRho cells migrate normally in 1/4 cases. Control cells of the CA condition migrate normally in 5/5 cases; CACdc42 cells migrate normally in 3/6; CARho cells migrate normally in 1/4 cases. Controls were injected with UAS:mCherry plasmid. All embryos were siblings from the same batch of injection. In images, the otic vesicle is outlined with a faint grey dashed ellipse. Tracked cells are pseudocoloured in magenta and tracks in white. Scale bars: 20 µm. See also Movie 8. ns, not significant.

We first overexpressed the DN form of Rac1a by injecting into one-cell-stage embryos the UAS: DNRac1a-F2A-GFP Tol2 construct (Hanovice et al., 2016). We then tracked, over a period of 10 h, control cells under CRISPR Eraser conditions and Rac1a inhibited cells expressing GFP (Fig. 3A versus Fig. 3A′; Movie 7). Compared with control cells, DNRac1a cells showed a reduced migration distance irrespective of their initial position (lateral, medial or posteroventral; compare white tracks from pseudocoloured magenta cells in Fig. 3A versus Figs 3A′ and 3D). All tracks in Fig. 3D were plotted using DiPER (Gorelik and Gautreau, 2014), as well as four individual tracks as examples (*n*=25 control cells and *n*=30 DNRac1 cells, from 7 and 14 embryos, respectively). Fig. 3E represents a 95% confidence interval of 2D spatial dispersion of NBs considering the final position of every NB in control (blue dots) and DNRac1a (orange dots) conditions after normalizing the tracks to origin (Breau et al., 2017; Gorelik and Gautreau, 2014). In summary, Fig. 3E shows the covered area (ellipses) or dispersion of NBs in control versus DNRac1a conditions, assuming they all depart from the same point (see ‘2D dispersion analysis’ section in Materials and Methods). Non-overlapping regions and different ellipse centroids (black dots in Fig. 3E) revealed differences in the dispersion of NBs between both conditions. In addition, there was a significantly reduced distance in the *x*-axis and a reduced effective distance travelled in DNRac1a versus control conditions (Fig. 3F, ticks in the *x*-axis represent 10 µm increases). The effective distance travelled by cells is the straight line from the initial to the final position.

DNRac1a-expressing NBs had similar quantified velocities (Fig. 3G, left graph) and net directionality (Fig. S6, wind rose plots) than control cells. However, the migrated distance was reduced in DNRac1a conditions (Fig. 3E,F). This indicates that the persistence of directed migration in DN-expressing NBs is impaired. In summary, DNRac1a-expressing NBs still move and at similar rate velocities to controls but lose persistence in directed migration. When we imaged a few delaminating NBs expressing DNRac1, Pard3 still relocated during delamination, but these cells did not fully delaminate (Fig. S2).

We extended the analysis by abrogating (DN) and increasing (CA) the function of the RhoGTPases Cdc42 and Rho1 in otic NBs (Fig. 3B,C; Movie 8). In these conditions, we found that NBs also have altered migratory capacities (Fig. 3C, asterisks; quantification in Fig. S7).

Altogether, our experiments blocking and activating RhoGTPases reveal for the first time that the RhoGTPase pathway is required for a directed and active migration of delaminated otic NBs. This suggests that otic NBs engage in active and complex behaviours to organize the SAG, rather than undergoing passive and bulk organization.

### NB display a characteristic migratory profile to a coalescence region

**Fig. 4.**
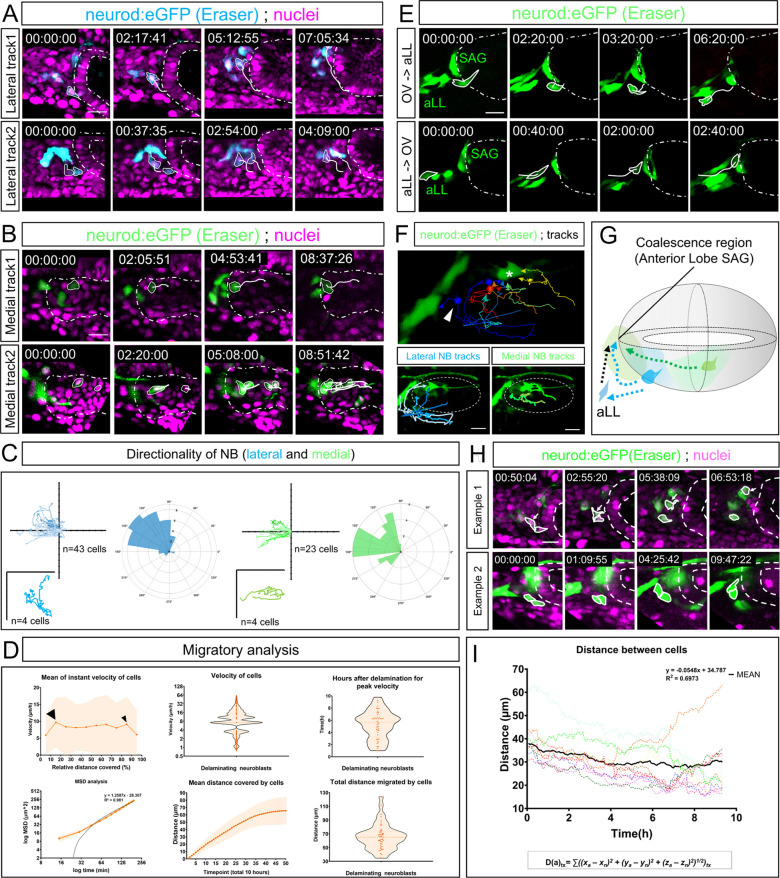
**Delaminating NBs directionally migrate towards a common coalescence region.** (A) Two tracks (white lines) of delaminated NBs from lateral domains of the otic neurogenic region. (B) Two tracks (white lines) of delaminated NBs from medioventral domains of the otic neurogenic region. The otic vesicle contour is delineated with a white dashed line. See also Fig. S8 and associated Movies 9 and 10. (C) Summary tracks using DiPER normalized to the origin and directionality rose plots (cyan for lateral and green for medioventral) of delaminating NBs (Breau et al., 2017; Gorelik and Gautreau, 2014) showing that migration of NBs is directed. Ticks in axes represent 10 µm increases. (D) Graphs of quantified data of NB migratory properties. Black arrowheads depict maximum velocities. Individual data shown in dark orange dots. Horizontal line is the median. Light orange shadows represent the s.d. For a separate analysis of lateral and medial delaminating cells, see Fig. S8. (E) Top panels show an otic delaminating NB (outlined in white) migrating towards the aLL. Lower panels show an NB (outlined in white) migrating towards the SAG. (F) Tracking several NBs in the same embryo shows aggregation towards a common region (asterisk) irrespective of NB origin inside the otic neuroepithelia. White arrowhead indicates an otic NB attracted to the aLL. (G) Graphic summary of migratory paths undertaken by NBs. Lateral NB shown in blue, medial NB shown in green. Dotted lines represent the migratory paths. Black dotted line shows aLL NB migrating to the SAG. (H) Qualitative examples of non-collective and collective migration. Otic epithelium encircled with dashed line. Migrating NBs outlined with white line. (I) Plotting several cases summing the distance of one NB with respect to its neighbours across times shows a negative overall regression, which is indicative of distance shortening between cells, thus indicating aggregation. Otic vesicles are depicted inside white dashed lines. Images in A and H are from the same raw data as Movies 9 and 11, but with different contrast enhancements to the images to show tracking of individual or contiguous cells. Anterior is always to the left and posterior to the right. aLL, anterior Lateral Line. Scale bars: 20 µm.

Based on our findings that otic NBs actively migrate, we then investigated which migratory paths are followed by NBs. Using CRISPR Eraser in *Tg(neurod:eGFP)* and photoconverted cells in *Tg(neurod:kikume)* backgrounds, we tracked delaminating NBs starting at their initial point of delamination in the neurogenic domain for a period of 10-12 h from the delamination peak of 20-24 hpf until 34-36 hpf. NBs delaminating from the anterolateral neurogenic domain followed a longer migratory path and moved from lateral to medial positions (Fig. 4A, cyan; Movie 9). By contrast, NBs delaminating from posteromedial regions migrated anteriorly to the same region between the otic vesicle and the hindbrain (Fig. 4B, green; Movie 10). Wind rose plots of the net vector directionality confirmed the directed anteromedial migration of delaminated NBs (Fig. 4C, colour coding preserved). Tracking of many migrating NBs [*n*=66 (43 lateral and 23 medial) cells from 40 embryos] highlighted their directional migration towards a region just anterior and medial to the otic vesicle and attached to the hindbrain wall at rhombomere 4 (Fig. 4F, asterisk; Fig. 4G). We found that migration and coalescence to this region contributes to the growth of the anterior lobe of the SAG (Fig. 4G). We named this region the coalescence region. Although it has been suggested that medial delaminated NBs remain there and position in the posterior lobe (Dyballa et al., 2017), our individual tracking of medial delaminated NBs showed that they also migrate and coalesce anteriorly.

The time elapsed from delamination until NBs reach the coalescence region, which normally coincides after a rapid movement of peak velocity, was approximately 6 h (Fig. 4D, smaller black arrowhead; Fig. 4D, upper-right panel; Fig. S8C,C′,E, black arrowheads).

Plotting the mean of instant velocities of NBs against normalized distance covered revealed a slight increase in velocity just right after delamination. Subsequently, NBs displayed a fluctuating walking behaviour (Fig. 4D, larger black arrowhead). NBs interchanged steady moments with pulses of movement, which is indicative of the previously reported active migration of cells, and coincides with the three different phases of behaviours described in Fig. 2A,B. Finally, NBs increased their velocity until they reached a region where they stopped and coalesced (Fig. 4D, small black arrowhead; Fig. S8 distinguishes lateral and medial delaminating NB behaviours). The quantified maximum velocity of migration was 61.2 µm/h (Fig. 4D), the maximum distance covered was 124 µm and the mean distance migrated 66 µm. Mean square displacement analysis of the data with DiPER (Gorelik and Gautreau, 2014) showed a slope (black line in Fig. 4D, lower left panel) of the linear fit that was greater than 1 (α>1), which is indicative of directed motion. For further detailed and separated analysis between more lateral and more medial NBs, see Fig. S8.

Surprisingly, in some instances, we also observed otic delaminating NBs being able to incorporate into the anterior lateral line (aLL) ganglion and vice versa, suggesting that both neuronal tissues are plastic, receiving and sending a few neuronal progenitors (Fig. 4E; 4F, white arrowhead) the fates of which are not fully determined yet.

We previously observed that delamination of NBs can be collective, which made us curious to investigate whether migration was also collective. Collective migration is defined as a group of cells that keep cell–cell contacts, have group polarization and exhibit a coordinated behaviour relevant for the proper organization of the tissue (Le et al., 2022). To address this question, we followed in several cases two touching NBs and their neighbours. As shown in Fig. 4H, delaminated NBs can migrate non-collectively (Fig. 4H, example 1, white arrow; Movie 11) interrupting their physical contact and separating NBs from each other. In other cases, two touching cells migrated together (Fig. 4H, example 2; Movie 12), maintaining their physical contacts during the whole imaging period. Therefore, the data suggest that NB migration within the SAG does not require permanent NB cell–cell contact per se.

Nevertheless, our previous results indicated a common pattern of migration to the coalescence region, resulting in a migration in streams. When analysing and plotting the distance between several NBs against the rest of the neighbour cells across time, a negative linear regression appeared, confirming NB coalescence as a common feature for migrating NBs (Fig. 4I, black line).

In summary, NBs delaminating from different regions of the neurogenic domain migrate and aggregate around a defined region, which we defined as the coalescence region. The coalescence of NBs in this region gives rise to the anterior lobe of the SAG (Movie 13).

### Pioneer SAG neurons populating the coalescence region are required for NB migration and SAG organization

**Fig. 5.**
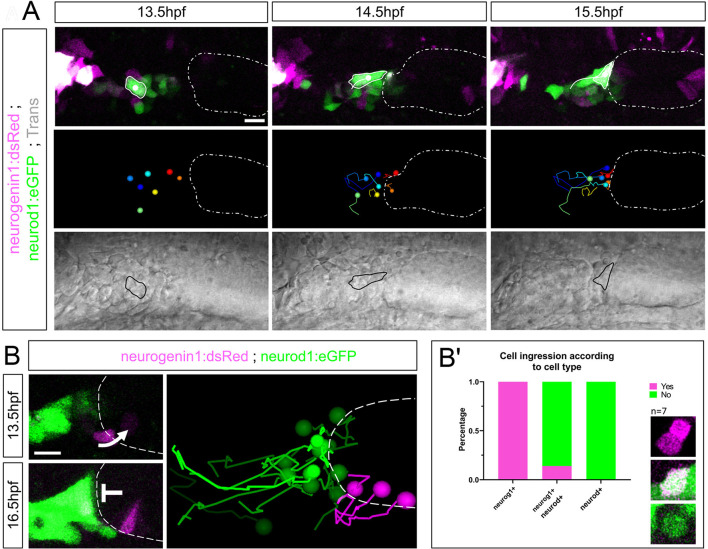
**Pioneer SAG neurons of extra-otic origin initially populate the coalescence region.** (A) Extra-otic pioneer SAG neurons are specified anterior to the otic placode (white dashed oval) and posterior to the trigeminal ganglion (magenta signal in left corner) at around 13 hpf. Pioneer SAG neurons migrate and initially populate the coalescence region in anterior locations of the otic placode. Migrating cells outlined in top and lower panels. Medial panels show different cell trajectories towards the otic vesicle (dashed line). (B) Pioneer SAG neurons are Neurod^+^ cells that do not ingress into the otic epithelium. Ingression takes place at 13.5 hpf (arrow) but not later (inhibitory symbol). Otic vesicle outlined with dashed line. Right panel shows ingressing cells in magenta and non-ingressing cells in green. (B′) Quantification of the number of cells ingressing or not depending on gene expression. Scale bars: 20 µm (A); 10 µm (B).

The coalescence region where delaminating NBs aggregate is populated by bright *neurod-*expressing cells. To assess the origin of these cells, we performed time-lapse analysis at 13.5 hpf, before otic delamination begins. A group of few scattered cells expressing either *neurogenin 1*, *neurod* or both were detected anterior to otic placode territories and posterior to the trigeminal ganglion (Fig. 5A). These cells migrated to anterior regions of the otic vesicle (Fig. 5A, white dashed oval) and apposed the 4th rhombomere wall by 16 hpf, populating the location that will later become the coalescence region. From this group of cells, the subset of *neurogenin 1^+^-*only cells, observed with the reporter line *Tg(neurog1:dsRed)*, ingressed into the otic epithelium as shown previously by Hoijman et al. (2017), whereas the subset of *neurod^+^* cells remained outside (Fig. 5B,B′). We term this latter group pioneer SAG neurons, because they are the first ones populating the prospective SAG ganglion.

**Fig. 6.**
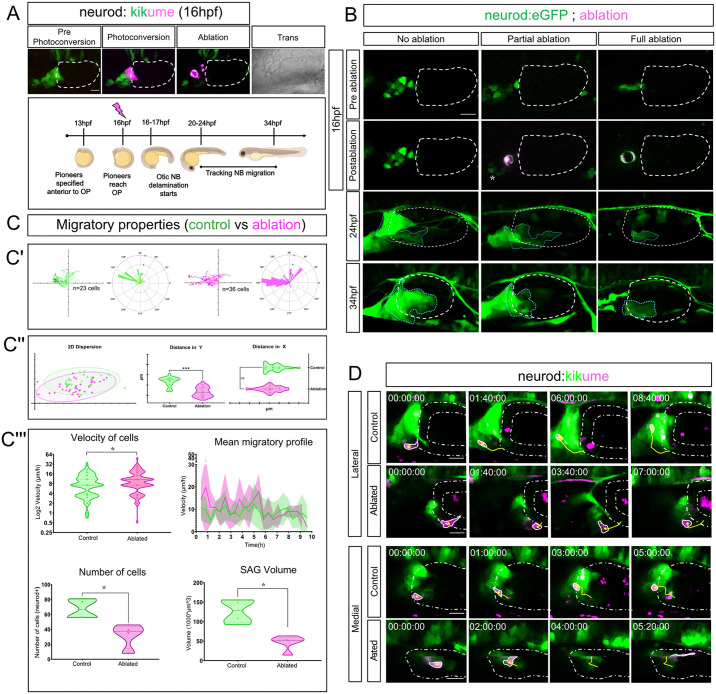
**Pioneer SAG neurons have a role in organizing the SAG.** (A) Scheme of experimental design. At 16 hpf, pioneer SAG neurons are photoconverted and subsequently photoablated. The absence of magenta photoconverted cells at later stages indicates complete cell ablation. SAG development was imaged from 20-24 hpf to 34 hpf. OP, otic placode. (B) After partial (asterisk) or total ablation of Neurod^+^ cells anterior to the otic placode at 16 hpf, an altered shape of the SAG and an apparently reduced number of NBs can already be observed at 24 hpf. At 34 hpf, the formation of the posterior lobe is abrogated in the ablated conditions (blue dotted line; see also Fig. S10). The experiment was replicated three times. *n* control=4+3+6; *n* ablated condition=4+3+11 (*n*=number of embryos). (C) Migratory properties of otic NBs in control (green) or after ablation of pioneer SAG neurons (magenta) measured with DiPER (Breau et al., 2017; Gorelik and Gautreau, 2014). (C′) NB migration directionality and pathway are compromised in after pioneer SAG neuron ablation compared with controls. *n*=23 control cells, *n*=36 ablated condition cells, from 4 and 10 embryos, respectively for each condition. Each tick in axes represents a 15 µm increase. (C″) NB dispersion (95% confidence interval) according to the last timepoint position with tracks normalized to the origin in control (green dots) versus ablated (magenta dots) conditions. Non-overlapping regions illustrate the variance in dispersion, which is significant in the *y*-axis but non-significant in the *x*-axis (ns). Ticks in axes represent 10 µm. (C‴) NBs are able to migrate faster in the ablation condition. The mean migratory profile is not majorly affected, except that dispersion of velocity is increased in the ablated condition at initial stages of migration (Fig. S11 shows detailed individual migratory profiles). The number of cells populating the SAG is significantly reduced in the ablation condition, more than the number of cells ablated (Fig. S9A) and consequently SAG volume (Fig. S10). *N* number of embryos for this section (C‴) is 4 both for control and the ablated condition. *P<0.05, ***P<0.0005 (two-tailed Student's *t*-test). (D) Two examples of migration of NBs (photoconverted magenta cell, yellow tracks) between lateral and medial regions of the neurogenic domain. The otic vesicle is depicted within a white dashed line. Total control embryos *n*=6; total ablated embryos *n*=11 for this section (D). Scale bars: 20 µm.

**Fig. 7.**
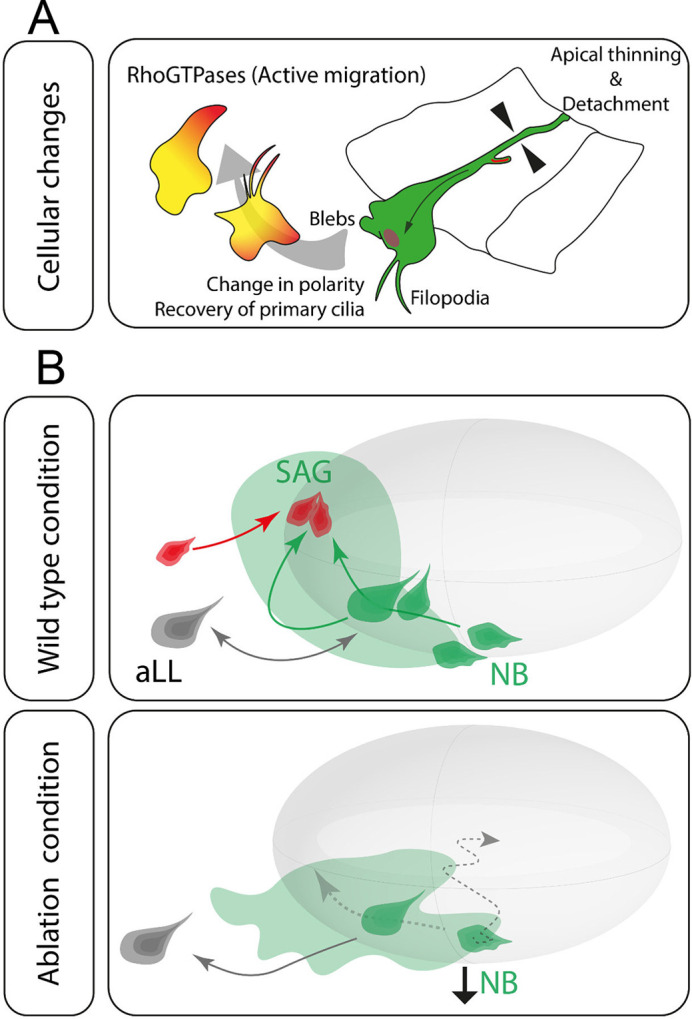
**Pioneer SAG neurons are required for SAG development.** (A) Summary of the cellular behaviours of otic NBs addressed in this study. (B) Lack of pioneer SAG neurons (red) disrupts SAG shape (light green) and NB migration (dark green cells and arrows). Anterior Lateral Line (aLL) NB depicted in grey.

To address whether pioneer SAG neurons have a role in migration and coalescence of NBs, at 16 hpf we photoconverted these cells in our *Tg(neurod:kikume)* line from green to magenta and then ablated them with two-photon microscopy (Fig. 6A). Several SAG defects were observed at later stages as a result of different experimental conditions, whether pioneer SAG neurons remained unablated, partially ablated (Fig. 6B, asterisk) or fully ablated (Fig. 6B; blue dotted line indicates SAG shape in *z*-projections of images). In ablated embryos, SAG shape was aberrant and the number of cells populating the SAG was reduced at 24 hpf (Fig. 6B,C‴; *n*=23 control cells, *n*=36 ablated condition cells, from 4 embryos and 10 embryos, respectively). At 34 hpf, the formation of the posterior lobe was abrogated (Fig. 6B). After tracking individual NBs when SAG pioneer neurons were missing, we found that the directionality patterns of migration was altered, as well as the dispersion of NBs (Fig. 6C′,C″; Movie 14; Figs S9-S11), which is probably the cause of the misshaped SAG. Fig. 6C″ shows, as in Fig. 3E, a 95% confidence interval of dispersion of NBs at their final position when normalized to the origin, comparing control (green) and ablated (magenta) conditions. Non-overlapping regions evidenced differences in NB dispersion, which are statistically significant according to *y-*axis distance (Fig. 6C″). Although the NB directionality pattern was less consistent, NBs in the ablated condition were capable of migrating faster (Fig. 6C‴, upper panels). Moreover, an absence of pioneer SAG neurons affects the number of NBs that form the SAG and, consequently, SAG volume was also reduced (Fig. 6C‴, lower panels; Fig. 7).

Altogether, our results point towards a fundamental role of pioneer SAG neurons in recruiting delaminated NBs and thus orchestrating the shape and growth of the SAG (Fig. 7).

## DISCUSSION

Little is known about the morphogenetic events of cranial sensory ganglia development at the cellular level in comparison with other model systems, such as epithelial tissues. In this work, we analyse for the first time at cellular resolution, the cell behaviours underlying NB delamination, migration and coalescence of the SAG.

### NB otic delamination as an EMT process

Whether otic NB delamination follows an EMT process has been under debate. Previous work suggests that sensory neuron delamination in cranial placodes is not an EMT event (Graham et al., 2007; Schlosser, 2008, 2010) because the Snail family of core-EMT transcription factors and RhoGTPases are not used in this process (Graham et al., 2007). Moreover, Sox9/10, important EMT regulators, were found to be absent in most *Xenopus* placodes (Schlosser, 2008, 2010). Additionally, the migratory capacity of cranial placode NBs seems to be diminished compared with that of neural crest (Schlosser, 2008). However, published gene expression data and our results argue the contrary, proposing that the delamination process of inner ear NBs has EMT characteristics.

Otic delaminating NBs express some EMT-associated genes, such as *sox9a/b* (Yan et al., 2005), *sox10* (Chiang et al., 2001; Dutton et al., 2009; Piloto and Schilling, 2010), the Spemann organizer gene *goosecoid* (Kantarci et al., 2016), *snai1b* (Léger and Brand, 2002; Zecca et al., 2015) and *cdh6* (Hans et al., 2013), which are known to be important for neural crest cell (NCC) migration (Clay and Halloran, 2014). Together with gene expression changes, EMT is accompanied by large cell-shape rearrangements, acquisition of extensive migratory capacity, apicobasal polarity changes and cell adhesion disassembly (Yang et al., 2020). Finally, otic NBs are still not postmitotic (Camarero et al., 2003; Hoijman et al., 2017), meaning delamination is not strictly of neurons, as it is in other placodes (Graham et al., 2007).

We show here the changes in cytoarchitectural dynamics, apical determinants and primary cilia relocalization during otic NB delamination. The sequence of cell shape changes involves an apical thinning, with most cytoplasm concentrating basally, and an eventual apical detachment from the lumen, in which some cell membrane leftovers remain, resembling neural tube delamination (Das and Storey, 2014). There is also extensive basal blebbing, lateral and basal filopodia extensions and final NB translocation out of the epithelium. Blebbing is an important mechanism to facilitate basal lamina removal and EMT in zebrafish NCCs (Berndt et al., 2008; Font-Noguera et al., 2021; Paluch and Raz, 2013). However, it is not yet clear in our tissue whether blebbing occurs as an active property for facilitating delamination/migration or a passive event due to forces from neighbouring cells and loss of basal lamina and membrane integrity (Schick and Raz, 2022).

Imaging of the apical protein Pard3 localization during otic delamination has revealed that Pard3 is partially kept in the apical membrane abscised particle, whereas a different fraction travels basally in the hinge point of apical cytoplasmatic thinning, suggesting it might provide information for the apical thinning process. In our system, compared with the laterality organ of zebrafish (Pulgar et al., 2021), partial delamination does not occur, but once NBs are out of the epithelium, Pard3 is dynamically rearranged but retained, similarly to zebrafish heart trabeculation (Jiménez-Amilburu et al., 2016). Pard3 maintenance in pseudo-mesenchymal NBs suggest that Pard3 could establish a new polarity axis in migratory cells or inform where neurites must grow, as shown in some mechanosensory neurons (Lee et al., 2021). In NCCs, relocalization of the centrosome on the other side of the nucleus during EMT results in a polarity reversal and is responsible for cell dispersal when it becomes mesenchymal (Burute et al., 2017). Moreover, Pard3 localization in cell–cell contacts during NCC migration promotes microtubule disassembly (Moore et al., 2013).

In chick, actin-based apical constriction leaves an apical abscised part containing the primary cilia membrane, but the centrosome remains in the delaminating cytosol (Das and Storey, 2014; Kasioulis et al., 2017). In our delaminating NBs, we observe internalization of the primary cilium. The fact that the primary cilium is not dismantled or kept on the abscised membrane might favour rapid re-establishment of polarity once delaminated NBs are outside the otic epithelium. It remains open whether there is passive apical membrane stretching and shearing while recovering apical proteins and primary cilium or whether, alternatively, there is active apical cell-autonomous cytoskeletal rearrangement, our data suggesting the former because of myosin contractility at the apical domain of the delaminating cell. It is known that Cdh6 and RhoA in apical domains lead to apical constriction in NCCs engaged in delamination *in vivo* (Clay and Halloran, 2014). In summary, data from our experiments and the literature suggest that otic NBs undergo an EMT process when delaminating.

### NBs as active entities to organize the SAG

Once outside the otic neuroepithelia, otic NBs acquire a much variable and rounded shape. We have shown that delaminating NB actively engage into complex migratory behaviours in a RhoGTPase-dependent manner. This suggests that NB migratory capacity is more constrained by neighbouring tissues and signalling than by inherent low migratory capacity (Graham et al., 2007; Schlosser, 2008). Moreover, NCCs maintain SAG aggregation through contact, but are not implicated in giving migratory cues to NBs (Sandell et al., 2014; Zecca et al., 2015). In NCCs, reducing Rac1, and consequently reducing lamellipodia extensions, affects NCC migration but delamination still occurs. This indicates that these protrusive extensions are required to generate a migratory force in NCCs, and thus large protrusions might be required in our tissue for the same purpose.

In collective migration, cells in a group move as a coherent entity with some cell reorganizations (Reig et al., 2014). In NCC tissue, E-cadherin (Cdh1) helps to maintain stream migration and Pard3–N-cadherin (Cdh2) interactions avoid crowding by contact inhibition of locomotion (Clay and Halloran, 2014; Moore et al., 2013; Priya and Yap, 2015; Scarpa et al., 2015). In our study, we find that two adjacent migrating NBs can detach from each other and migrate independently in a non-collective manner, although all cells maintain certain directional coherence. The extent to which cell–cell interactions are required for SAG migration remains to be explored. It is known that *itg5a* is required for the correct aggregation of epibranchial placodes (Bhat and Riley, 2011), maintaining tissue segregation from other placodes and cohesiveness within the tissue, but whether other integrins are also relevant for otic NB cohesiveness and migration is unknown.

Another key finding of this work is that delaminated NBs engage in active migration towards a coalescence region, irrespective of their origin location inside the neurogenic domain and their delamination position. Thus, when NBs delaminate they do not remain in the same location, but rather migrate and coalesce anteriorly. This suggests that the SAG becomes organized in a much more complex manner than previously assumed according to genetically encoded information.

### The posterior placodal area provides pioneer SAG neurons as organizing centres for SAG assembly

Neurogenic cells from the posterior placodal area (PPA) were known to exist but thought to contribute exclusively to aLL or the otic placode (Andermann et al., 2002). Here, we show for the first time how PPA neuronal-specified cells can contribute also to the SAG. These cells have a pioneer role over otic delaminated NBs and could also have a pioneer role in the aLL. In addition, we also observe that, in some cases, delaminated NBs can integrate into the aLL and vice versa. Thus, at very early stages, neurogenic cells of the PPA are still not fully committed and are interchangeable between aLL and SAG, in accordance to the hypothesis put forward by Abelló and Alsina (2007). The signals that help pioneer SAG neurons to become positioned anterior to the otic vesicle are unknown. The SDF1 (Cxcl12)/Cxcr4 system has a role in lateral line primordium migration; however, SDF1 has not been reported to be expressed in the otic placode. Molecules from the hindbrain could also play a role driving pioneer SAG neuronal population migration. Finally, chase and run interactions such as the ones described between NCC and placodes (Theveneau et al., 2013) were discarded owing to time window constraints as pioneer SAG neurons migrate before NCCs invade the tissue. NCCs are likely to have a later role in maintaining SAG cohesiveness as suggested (Begbie and Graham, 2001; Steventon et al., 2014; Zecca et al., 2015).

Pioneer neurons and tracts were discovered in seminal studies in *Drosophila* (Harrison, 1959) and recent studies show that they are required for other neuronal body positioning (Karkali et al., 2023). In zebrafish, a group of pioneer neurons is required for correct migration of later facial branchiomotor neurons (Wanner and Prince, 2013). Here, we provide evidence of pioneer SAG neurons of non-otic origin that, by positioning adjacent to the otic vesicle, also have a role in the migration of delaminated otic NBs and the organization of the anterior SAG lobe.

In summary, the current work identifies a group of pioneer neurons that guide NB migration and coalescence and, ultimately, are key in shaping the ganglion. This knowledge will expand our understanding on the development of cranial ganglia, in which pioneer neurons prefigure the definitive architecture and/or location of a neuronal tissue.

## MATERIALS AND METHODS

### Fish maintenance and husbandry of transgenic lines

Zebrafish embryos and adults were maintained and handled according to standard procedures at the aquatic facility of the Parc de Recerca Biomèdica de Barcelona (PRBB), in compliance with the guidelines of the European Community Directive and the Spanish legislation for the experimental use of animals and as previously described (Westerfield, 2000). Stable transgenic lines were kept by means of alternate outcross with wild type (AB/Tü) and incross, generation after generation. Expansion of the lines was carried out every 1.5-2 years. Embryos were kept under dark conditions at a temperature of either 23°C or 28.5°C in Danieau's solution.

For this study, we used the following lines: *TgBAC(neurod1:eGFP)nl1*, labelling specified neuroblasts and neurons (Obholzer et al., 2008); *Tg(neurod1:Gal4)* line (generated in the lab), in which Gal4 expression is driven by the *neurod1* promoter, insertion by CRISPR knock-in following published protocols (Auer et al., 2014a,b; Auer and Del Bene, 2014; Kimura et al., 2014) using the gbait plasmid from Kimura et al. (2014); *Tg(neurod1:Gal4; UAS: H2A-GFP)*, combining *Tg(neurod1:Gal4)* with injection of the Tol2 plasmid [UAS-H2A-GFP], kindly provided by Dr Jeroen Bakkers' Lab (Strate et al., 2015); *Tg(neurod1:kikume)*, generated in the lab by injecting a plasmid containing 5 kb *neurod* promoter upstream of the photoconvertible protein Kikume (plasmid kindly provided by Dr Katie Kindt's lab, National Institute on Deafness and Other Communication Disorders, USA); *Tg(neurogenin1:dsRedE nl6)*, which labels early specified neural progenitors (Drerup and Nechiporuk, 2013); *Tg(actb2:H2A-mCherry)* as a pan-nuclear marker, line provided by Dr Esteban Hoijman at Dr Verena Ruprecht's lab (CRG, Spain); *Tg(ubb:arl13b-EGFP)* labelling the primary cilia-related protein Arl13b (Austin-Tse et al., 2013); and *Tg(β-actin:myl12.1-eGFP)* labelling myosin II (Hoijman et al., 2015).

Embryos used later than 34 hpf were kept transparent by soaking them in embryo medium (Danieau's solution) with 1% 1-phenyl-2-thiourea (PTU) (Sigma-Aldrich) to inhibit pigment formation (Westerfield, 2000). This treatment did not affect development in controls. Embryos were staged as previously described (Kimmel et al., 1995). In all experimental conditions, the embryos of control and experimental conditions were siblings except when comparing DNRac1a with CRISPR Erasers.

### Microinjection

Long and very thin injecting needles were made in an electrophysiology puller (Sutter instruments model P-97) with the following protocol: *P*=200; HEAT=566; PULL=90; VEL=70; TIME=80. The tip of the needle was bevel broken using forceps and the needle was loaded with injecting solution. Embryos were injected with 1 or 2 nl into the cell or yolk in one-cell-stage embryos. Injections of mRNA ranged from 50 to 250 ng/µl. RNA synthesis was as described according to manufacturer's guidelines (AM1340, Invitrogen). sgRNA for GFPbait was as described by Gagnon et al. (2014).

Mosaic injections at the 32- to 64-cell stage were performed in the central cells according to fate map analysis (Strehlow et al., 1994; Strehlow and Gilbert, 1993) in order to maximize the probability of labelling the tissue of interest accurately.

### DAPI staining and cryosections

From a DAPI stock at 5 mg/ml we performed a 1:500 or 1:10,000 dilution in PBS with 0.1% Tween-20 (PBT 0.1%) for whole zebrafish up to 24 hpf or slides, respectively. Embryos or slides were submerged in the solution for 5 min and then washed for 5 min in PBT 0.1%. For slides, 20-µm-thick cryostat sections were adhered to specialized slides (StarFrost Objektträger Knittel glass). Cryosectioning was carried out as described by Taberner et al. (2020).

### Fiji processing

For visualization purposes only, images were non-linearly processed with Fiji plugins subtract background (50 pixels radius), smooth and/or gaussian 3D blur with *x*, *y*, *z* values of 1 or 2. Images were also linearly modified with the brightness/contrast tool and median filter with the same values within the same experimental batch. 3D drift correction was made according to nuclei, SAG and/or transilluminated otic vesicle with the ‘3D drift correction’ Fiji plugin (Movie 15) and enhanced drift correction options activated. Images are either *z*-projections from a stack or single planes.

### Confocal live-cell imaging

Anaesthetized zebrafish (42 µl tricaine per ml of water, from a stock of 400 mg tricaine powder in 100 ml H_2_O) were mounted on a 35 mm Ibidi μ-dish (81156 Ibidi) in 1% low melting point agarose 45° tilted from completely dorsal. Imaging of embryos was performed on an inverted Leica SP8 confocal system equipped with a 488 nm argon-ion laser (LASOS, 488 nm laser power 2.5 mW at back focal plane at 100% laser power), 405, 561, 633 laser diodes, motorized *xy* and *z*-galvo stage, one HyD and two PMT detectors and a HC PL APO 20×/0.75 IMM and CS2 objective (506191 and 506423, Leica). The 488 nm laser excitation depended on the experiment but was always kept the same across samples for the same and related experiments. For *in vivo* experiments, it ranged from 0.1 to 10%. For fixed embryos, it could increase up to 80%. The gain normally ranged between 600 and 900 Hz and off-set was never higher than 0.1%. Fluorescence excitation at 488 nm was collected between 500 and 543 nm, and fluorescence excitation at 561 nm was collected between 589 and 621 nm. Leica LUT setting2 was used to set a high-low mode, which was used for setting the pixel saturation limits (background pixel value 0=green; saturated pixel value 255=blue).

Frame sizes of 1024×512 or 1024×1024 at 8-bit were used. Scan speed ranged from 400 to 1000 Hz (normally 600 Hz). *z*-step sizes were system optimized according to Leica settings [an HC PL APO 20×/0.75 IMM objective (506191) typically gives a *z*-step size optimized at 1.19 µm with pinhole in airy1]. Software zoom factor was set at 2.5× for most of experiments. Bidirectional scanning was activated. Line average and frame average either 1 or 2 was used. Multi-position (Mark & Find) experiments included up to 35 embryos separated by a time frame no longer than 20 min, except where otherwise mentioned. To avoid cross-talk between fluorophores, sequential line acquisition was used. Cross-talk was checked *in silico* using FP base (https://www.fpbase.org/spectra/).

Tissue drift was considered and corrected. Otic tissue moved anteriorly at a rate of 4-10 µm/h. Movies of up to 12 h considered a total anterior drift of 120 µm maximum.

### Resonant scanning

To maximize temporal resolution time-lapse imaging of filopodia, the resonant mode (8000 Hz) of the Leica SP8 confocal microscope was used to achieve a high spatiotemporal resolution. Frames were captured at a time interval of 5 to 15 s. Optimal *z*-size according to the objective was used (normally 1.19 µm with pinhole airy1). Confocal stacks and movies were flattened by maximum projections using Fiji (ImageJ). The rest of the parameters remained the same as described in the ‘Confocal live-cell imaging’ section.

### Photoconversion

Photoconvertible transgenic embryos from *Tg(neurod:kikume)* were kept as much as possible away from light to avoid spurious photoconversion, although basal levels are always present in our hands. Circular or hands-free designed regions of interest (ROIs) the size of few cells were drawn in SP8 and SP5 inverted Leica microscope systems. In both microscopes, photoconversion was performed with a UV 405 laser line at 5 to 15% diode power upon ROI at 200 Hz during six scans and 3D ROI aiming the centre of the photoconverted region to avoid same level of photoconversion from upper or lower planes (although this was inevitable to some extent). Bidirectional laser scanning was used. A Leica 506191 objective (20×) was used in glycerol and PMT detectors were employed. Phototoxicity controls were performed as follows: laser power 100% at 200 Hz during several scans (at least six). Cells did not seem to be affected, at least within 6-8 h of recording.

### Photoablation

An SP5 inverted Leica Multiphoton confocal microscope was used with Mai Tai multiphoton activated [Mai Tai BB DeepSee (Spectra Physics) tunable (710-990 nm) pulsed laser], humidity 4%, temperature 20°C. A BS/RLD mirror and SP715 filter were used with a 910 nm laser at 42% power, at 20× zoom with an HC PL APO 20×/0.75 immersion objective (506191, Leica) because no ROIs can be used in this confocal in multiphoton mode. PMT was employed and the pinhole kept completely open (600 nm). Scans ranged from three to six until a bubble formed (indicating destroyed tissue). Scan speed was 200-400 Hz, frame size 1024×512, bidirectional scanning was on, with line and frame average 1.

### Generation of *Tg(neurod:Gal4)* line (two gRNAs) for CRISPR knock-in

CRISPR RNA (crRNA) sequences were selected using CHOP-CHOP (chopchop.cbu.uib.no) and ordered from Integrated DNA Technologies (IDT; 2 nmol) and 2-100 nmol trans-activating CRISPR (tracrRNA) was obtained from Integrated DNA Technologies. Cas9 protein was ordered from IDT (Alt-R™ S.p. Cas9 Nuclease V3, 100 µg, 1081058). crRNA and tracrRNA were resuspended to a concentration of 100 µM (20 µl) in IDTE buffer 1× (IDT, 11-05-01-14); 5 µl crRNA was mixed with 5 µl tracrRNA in 10 µl DUPLEX buffer IDT (IDT, 11-05-01-12), to obtain a final concentration of 25 µM of duplex guideRNA (dgRNA). The mixture was incubated for 5 min at 95°C and then cooled down to room temperature. Aliquots were stored at −20°C before use. The following mix was prepared: 0.86 µl RNase-free H_2_0 with 1 µl of total dgRNA, 1 µl of 250 ng/µl *gbait* single guide RNA (sgRNA) and 1.5 µl of 1 μg/µl Cas9. This mix is called RNP (ribonucleoprotein complex). The RNP was warmed up to 37°C for 10 min and 0.5 µl of the donor plasmid ‘gBait hsp70Gal4FF’ (Kimura et al., 2014) was added at 200 ng/µl to a final volume of 5 µl. The final conditions of this mixture were: total dgRNA=5.7 µM=200 ng/µl; *gbait* sgRNA=50 ng/µl; Cas9=300 ng/µl; donor plasmid=20 ng/µl. Subsequently, 1 nl of this mixture was injected into each embryo. The CRISPR knock-in protocol described above derives from modifications of previous protocols described in Auer et al. (2014a,b), Hoshijima et al. (2016, 2019), Kimura et al. (2014) and Thomas and Raible (2019).

### Genotyping fish from the *Tg(neurod:Gal4)* line

*Tg(neurod:Gal4)* fish where either phenotyped by crossing with the UAS:Kaede or other UAS:reporter line, or by fin clipping and PCR genotyping with the following protocol. Anaesthetized adult zebrafish (42 µl tricaine per ml of water, from a stock of 400 mg tricaine powder in 100 ml H_2_O) were fin clipped. After that, genomic DNA extraction was performed using an N-Amp extraction kit (XNAT2 Extract-N-Amp Tissue PCR Kit XNAT2-1KT). PCR protocol was as follows: forward primer Gal4FF: GCAGGCTGAAGAAGCTGAAG; reverse primer Gal4FF: GGAAGATCAGCAGGAACAGC; 35 cycles of 94°C 3 min; 96°C 10 s; 57°C 15 s; 72°C 30 s; 72°C 10 min; hold at 4°C. The resulting product was 178 bp.

### Tol2 injections [RhoGTPases, *Tg(neurod:kikume)* stable line generation]

For Tol2 injections, 1 µl of *Tol2* mRNA at 175 ng/µl was mixed with 3 µl of plasmid at 50 ng/µl and 6 µl of dH_2_0 (final volume=10 µl) and 1 nl was injected into the cell (giving an injected quantity of 15 pg of plasmid and 17.5 pg of *Tol2* mRNA). For transient expression experiments, embryos were kept until experimental use. For stable line generation, positive embryos were taken to the fish facility at 5 days post-fertlization.

### CRISPR eraser

To retain eGFP in just a few cells, we designed CRISPR Eraser. This methodology consists of not perfectly efficient Cas9 cutting and frameshift of eGFP reporter in a given transgenic line. To perform this protocol, 1.5 µl of Cas9 at 1 µg/µl (62 µM) was mixed with 1 µl of *gbait* sgRNA at 250 ng/µl and 2.5 µl of RNase-free H_2_O to give a final volume of 5 µl and concentrations of sgRNA and Cas9 of 50 ng/µl and 300 ng/µl (6 µM), respectively. One-cell-stage embryos were injected with 1 nl of this solution into the cell or yolk (yolk injections give more labelled cells). Injected quantity was 50 pg sgRNA and 300 pg Cas9. It is important that the transgenic line contains the GFPbait sequence designed by Kimura et al. (2014) for proper cutting. This can be confirmed by sequencing or *in vitro* assay of PCR product cutting. GFPbait sequence was GGCGAGGGCGATGCCACCTACGG.

### Quantifications

Nuclei were counted manually and measurements were performed with Fiji (ImageJ V1.5) from a defined ROI. When ROIs were used, the same area was used to compare samples. Cellular tracks were manually performed using TrackMate (Tinevez et al., 2017). Directionality analysis and plot to origin tracks was performed using DiPER (Gorelik and Gautreau, 2014) according to Breau et al. (2017).

### 2D dispersion analysis

From merged-to-origin migratory plots (Figs 3D, 4C and 6C′) obtained with DiPER (Gorelik and Gautreau, 2014), we extracted the relative spatial position in *xy* at the endpoint of the recording of each NB. The final relative position in *xy* of each NB was plotted in RStudio (4.2.0) using the SDD function from the SIBER library obtained from https://cran.r-project.org/, based on the pipeline given in https://cran.r-project.org/web/packages/SIBER/vignettes/Introduction-to-SIBER.html (Jackson et al., 2011). The SIBER library fits bi-variate ellipses to spatial data using Bayesian inference. Ellipsoid data accounting for the 95% confidence interval of data dispersion and ellipse centroid was retrieved and plotted with final relative position in *xy* of each NB in Microsoft Excel.

### Kymographs

A line of 1- to 15-pixel thickness was drawn on 4D recordings (*x*, *y*, *z*, *t*) in our ROI, and the ‘Kymograph’ option in Fiji was used to deploy a spatial section (*x*-axis) of an image with temporal resolution (*y*-axis). Data can be interpreted as fluorescent signal crossing the drawn section as we move in time (*y*-axis). Graphically, time increments in the *y*-axis represent the spatial thickness of the drawn line.

### Statistical analysis and plots

All data were first tested for normal distribution using the Kolmogorov–Smirnov test and the Levene's test for homogeneity of variances. For two-group comparisons, two-tailed Student's *t*-test was used for parametric data or Mann–Whitney *U*-test for non-parametric data. Values are expressed as mean±s.e.m., mean ±s.d. or median. Graphs were generated using GraphPad Prism 8 software.

The G*power3.1 (Erdfelder et al., 2009) program was used to infer *a priori* the sampling number needed to obtain statistically significant results from an expected phenotype of inferred penetrance.

## Supplementary Material

10.1242/develop.201824_sup1 Supplementary information
