# Supplementary material for: Pioneer statoacoustic neurons guide neuroblast behaviour during otic ganglion assembly
Source: Development. 2023 Nov 8;150(21):dev201824. doi: 10.1242/dev.201824 (PMC10651105; doi:10.1242/dev.201824)
Supplement: Supplementary information [file develop-150-201824-s1.pdf]

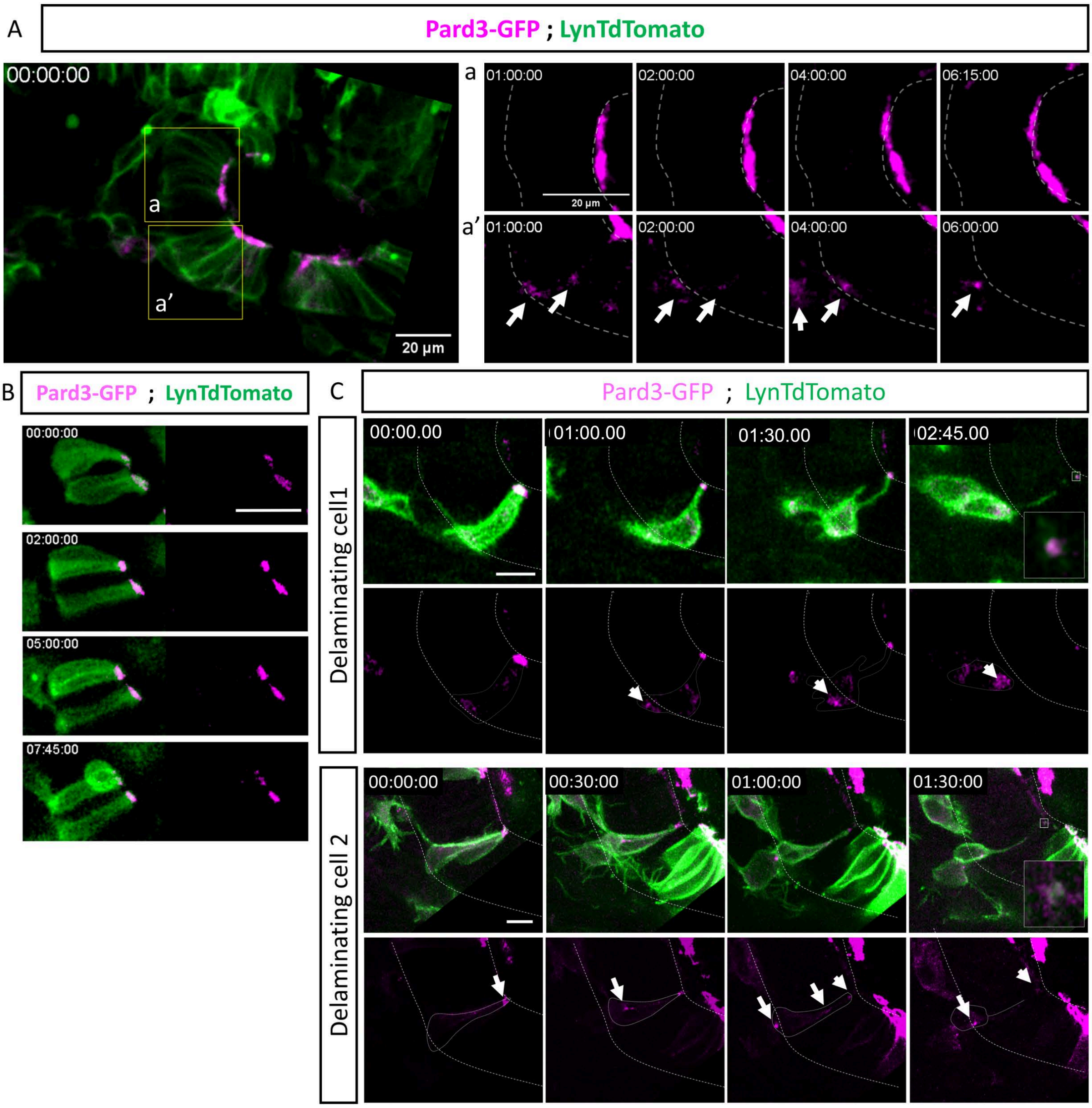

Fig. S1. Pard3 dynamics in non-delaminating and delaminating otic regions. A) Pard3 remains apical in non-delaminating otic regions (a) while it is relocated to basal side in delaminating regions (white arrowhead in a').

B) Membrane (LynTdTomato, green) and Pard3 labeling (magenta) in non-delaminating otic cells showing how these cells do not change their columnar shape and Pard3 is not relocated to basal sides (apical side to the right). C) Pard3 relocation (white arrows) in two more examples of delaminating NB (depicted with white dashed line) before and during delamination. The images shown in C (top) are from Movie 1, and are also depicted in Fig. 1C without Pard3-GFP fluorescence. Apical always to the right. Scale bar is 20µm when noted, otherwise is 10µm.

Development • Supplementary information

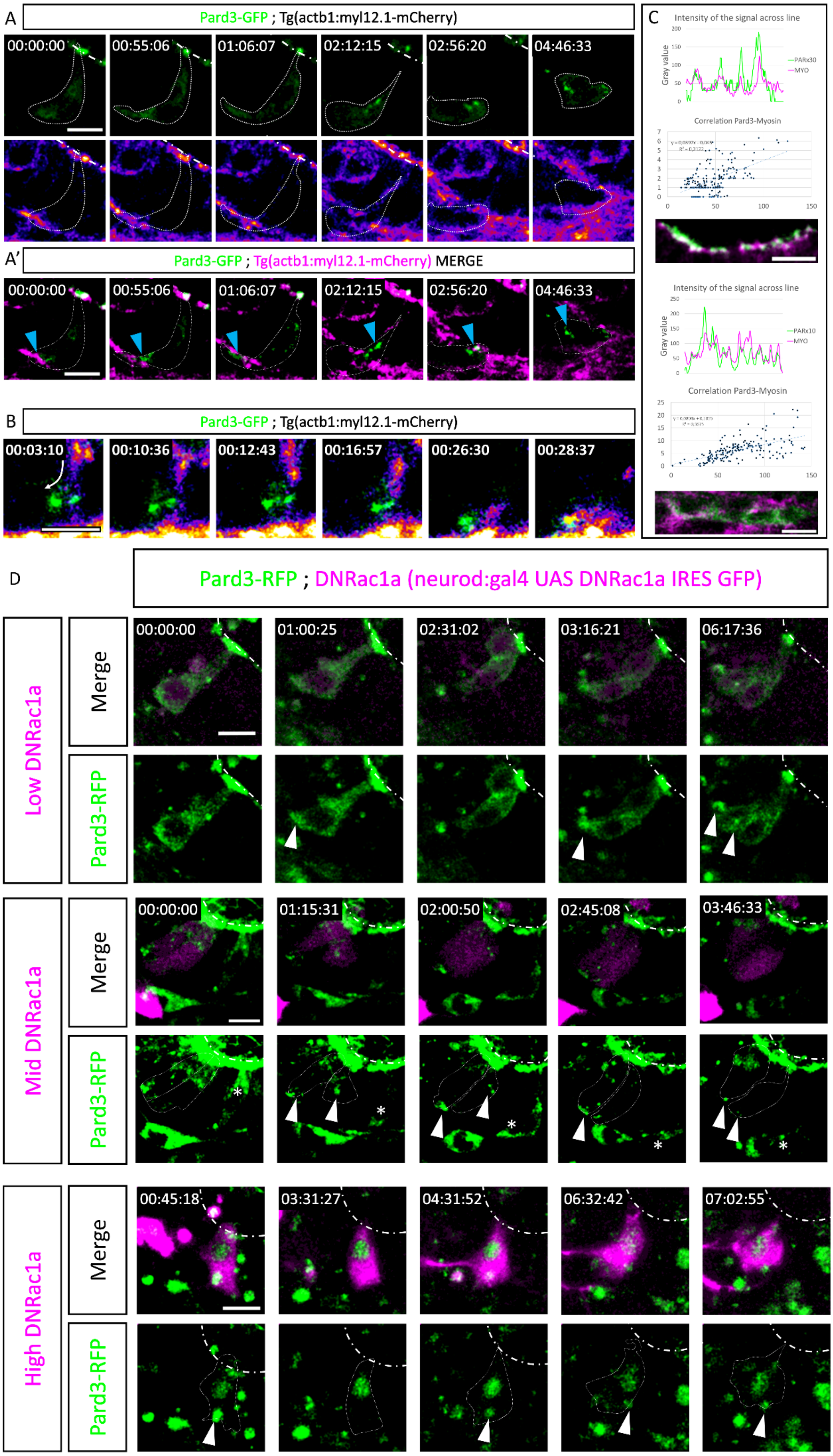

Development • Supplementary information

Fig. S2. Pard3 and myosin dynamics. A) Pard3 (green) relocates as previously described. Myosin enhanced signal (Fire LUT, 8-bit gray scale value 255 is yellow, while close to 0 is purple/black) is found in Pard3 vicinities, which can be appreciated together in panel A' (blue arrowheads). B) Higher temporal and spatial resolution experiment shows pard3 and myosin of a delaminating NB. Myosin follows Pard3 from behind (White arrow indicates movement of myosin in timelapse). C) Co-localisation analysis of Pard3 and myosin in apical domains (upper panels) and after delamination (lower panels), indicating that increased pard3 signal relates to increased myosin signal in pard3 vicinities. D) Rac1a RhoGTPase inhibition (DNRac1a, magenta) shows pard3 relocates (green, white arrowheads) to basal side of the cell. However, NB fail to delaminate. A delaminating NB not expressing DNRac1a (middle panel, asterisk [*]) shows pard3 relocation and delamination. Cells are outlined in white dashed line. Apical side depicted by white dashed and dotted line. Scale bars are 10µm.

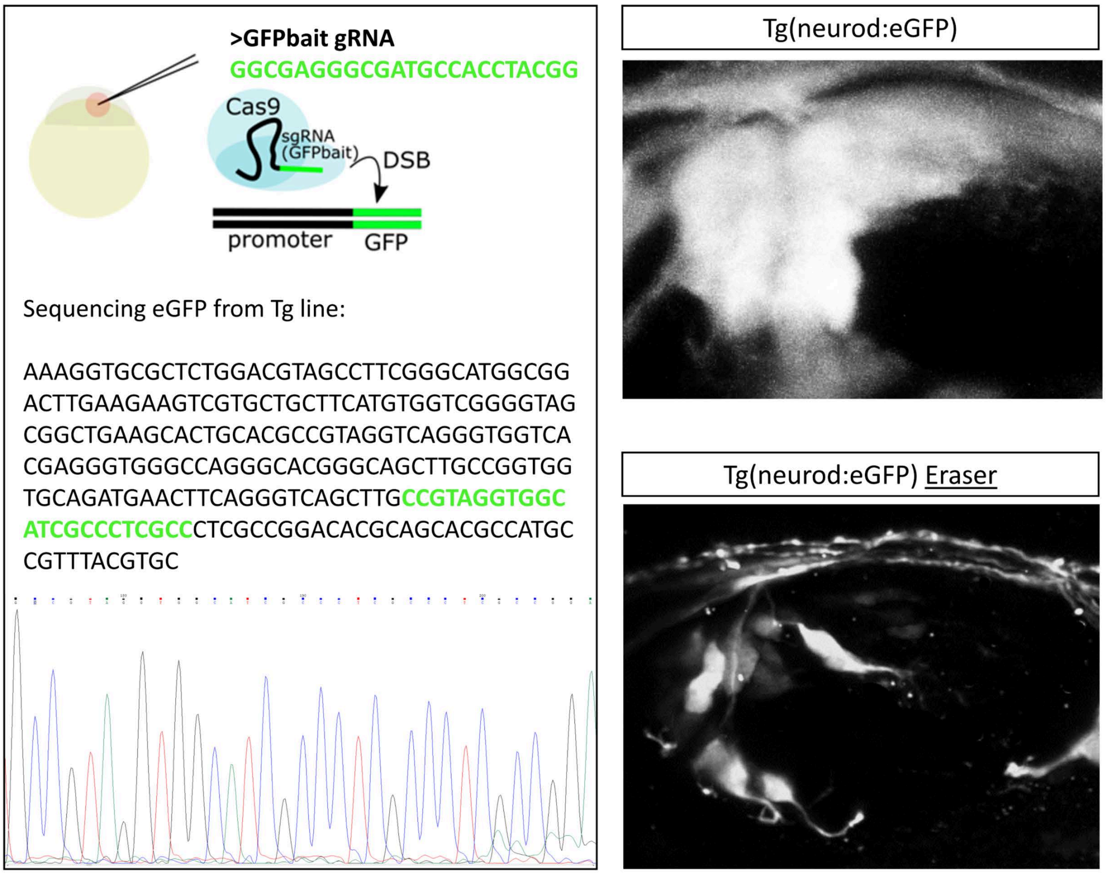

Fig. S3. CRISPR Eraser. Injection of a sgRNA (called GFPbait or *Gbait*) targeting endogenous GFP locus. GFP is maintained in just a few cells (lower right panel) compared to WT conditions (upper right panel). GFPbait sequence is present in the transgenic line eGFP locus, in our case Tg(neurod:eGFP). Anterior to the left, posterior to the right.

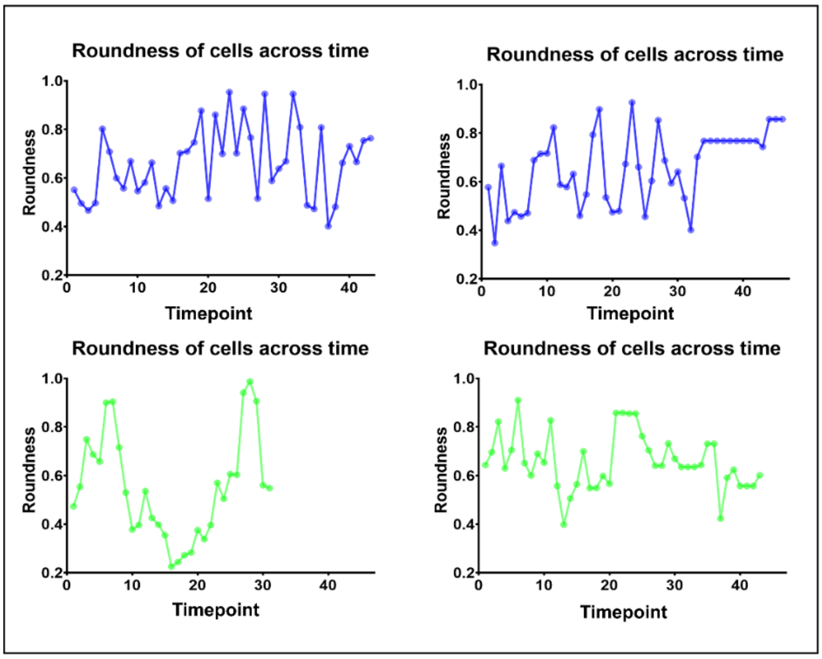

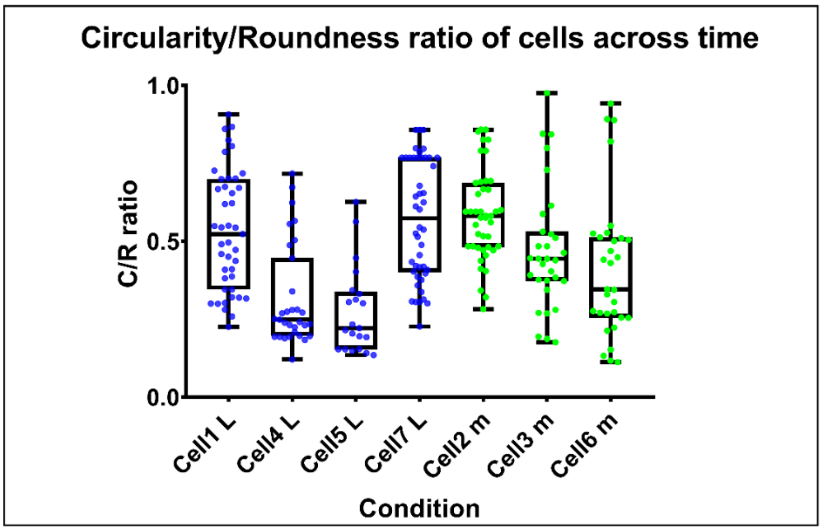

Fig. S4. Roundness and circularity of delaminated NB. Upper panel: 4 examples of NB roundness measurements through time. X axis refers to timepoint (1 image every 11-15 minutes). Y axis is roundness. Blue refers to lateral delaminating NB, while green refers medial delaminating NB. Lower panel: Circularity/Roundness ratio of 4 lateral and 3 medial delaminating NB. Measurements carried following the protocol from Krumblein and Sloss (1963).

Development • Supplementary information

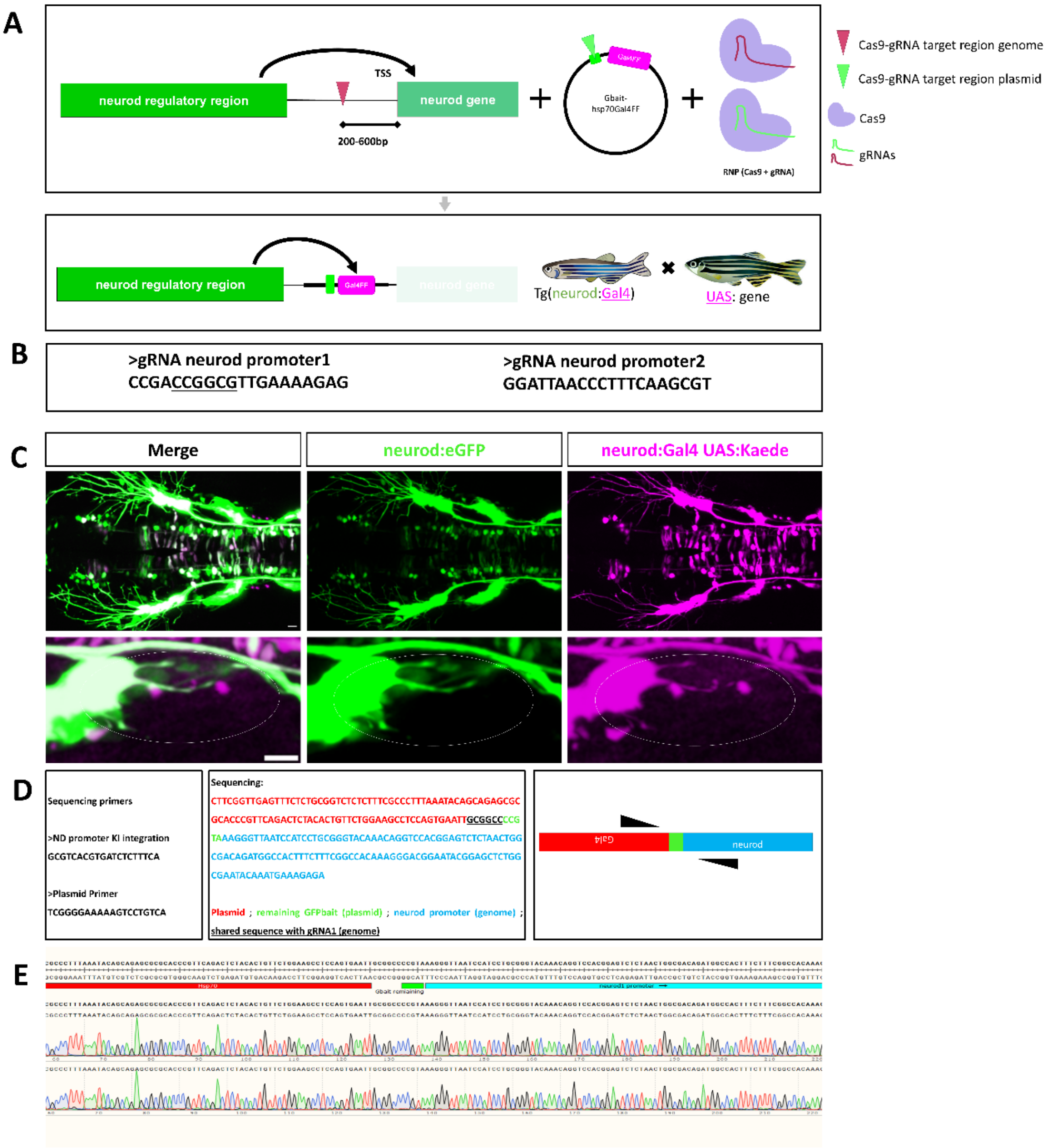

Fig. S5. Generation of Tg(neurod:Gal4) stable line. A) Scheme of injection. 200 to 600 upstream the transcription starting site (TSS), which is dowstream the neurod regulatory región, we choose a gRNA target region in the genome and a gRNA target region in the donor plasmid, which will be integrated. We injected the Cas9 protein with the gRNAs and the donor plasmid into the cell at 1-cell stage embryos. After correct integration, neurod regulatory region will regulate Gal4 expression. B) gRNA sequences targeting the genoma in our case. C) Comparative signal of the newly generated Tg(neurod:gal4) with a UAS:Kaede reporter compared to the already established and commonly used Tg(neurod:eGFP) line. Upper panels show a dorsal view of the hindbrain and cranial ganglia. Lower panels are insets of the otic domain (white oval). D) Primers used for sequencing Gal4 integration, sequence and schematic representation. E) Proof of correct integration by Sanger sequencing of a founder with both Fw and Rv primers. Scale bars are 20µm.

Development • Supplementary information

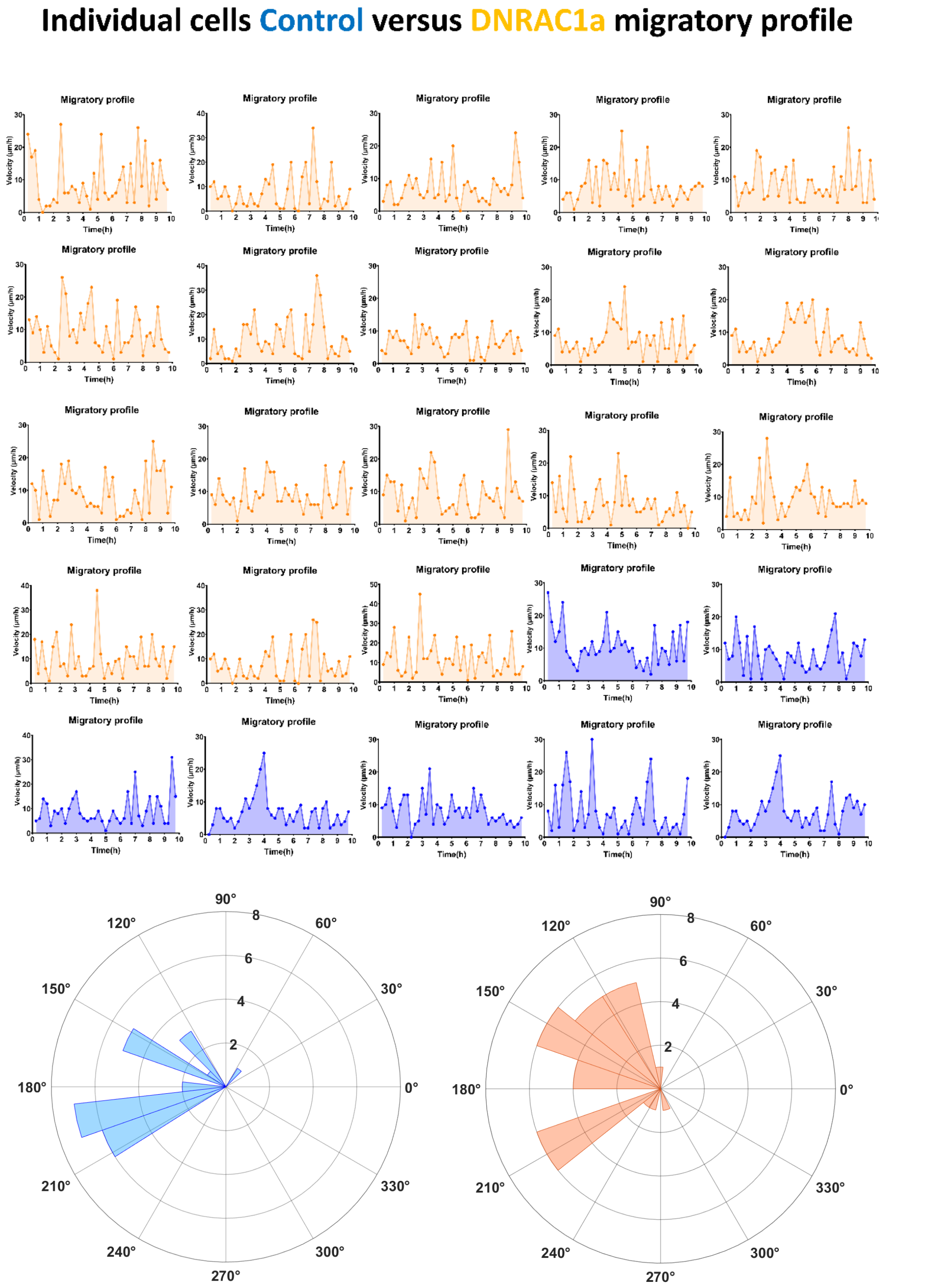

Fig. S6. Migratory profiles of some of the single NB in DNRac1a experiment and directionality wind rose plots. Orange are DNRac1a NB tracks, while blue are tracks of control NB. X axis, time (h). Y axis, velocity (µm/h). h: hours. Net directionality wind rose plots in control (blue) and DNRac1a (orange).

Development • Supplementary information

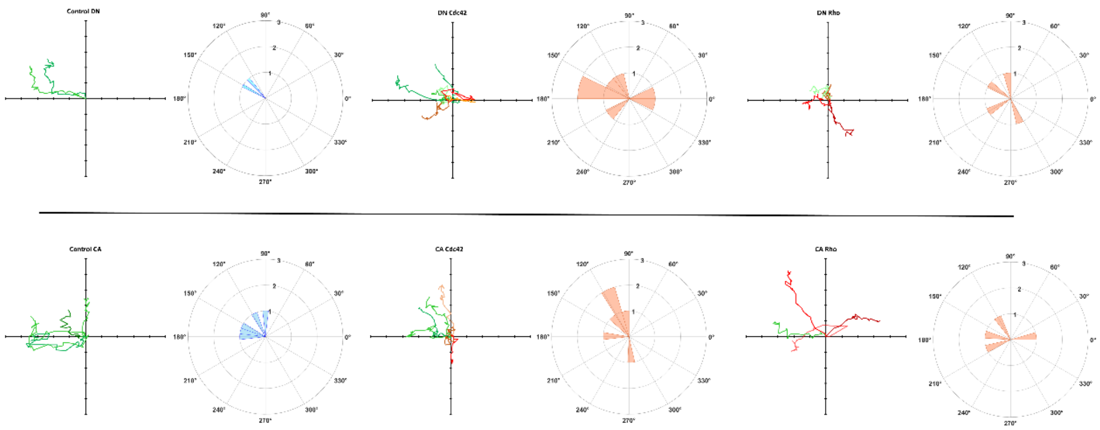

Fig. S7. NB migratory tracks and wind rose plots of directionality in (from left to right and from top to bottom) control, DN Cdc42, DN Rho, control, CA Cdc42 and CA Rho conditions. Adequate migration in migratory tracks is shown in green, while mild and strongly aberrant migration is shown in orange and red, respectively. Control wind rose plots are shown in blue, while altered RhoGTPases wind rose plots are shown in orange.

Development • Supplementary information

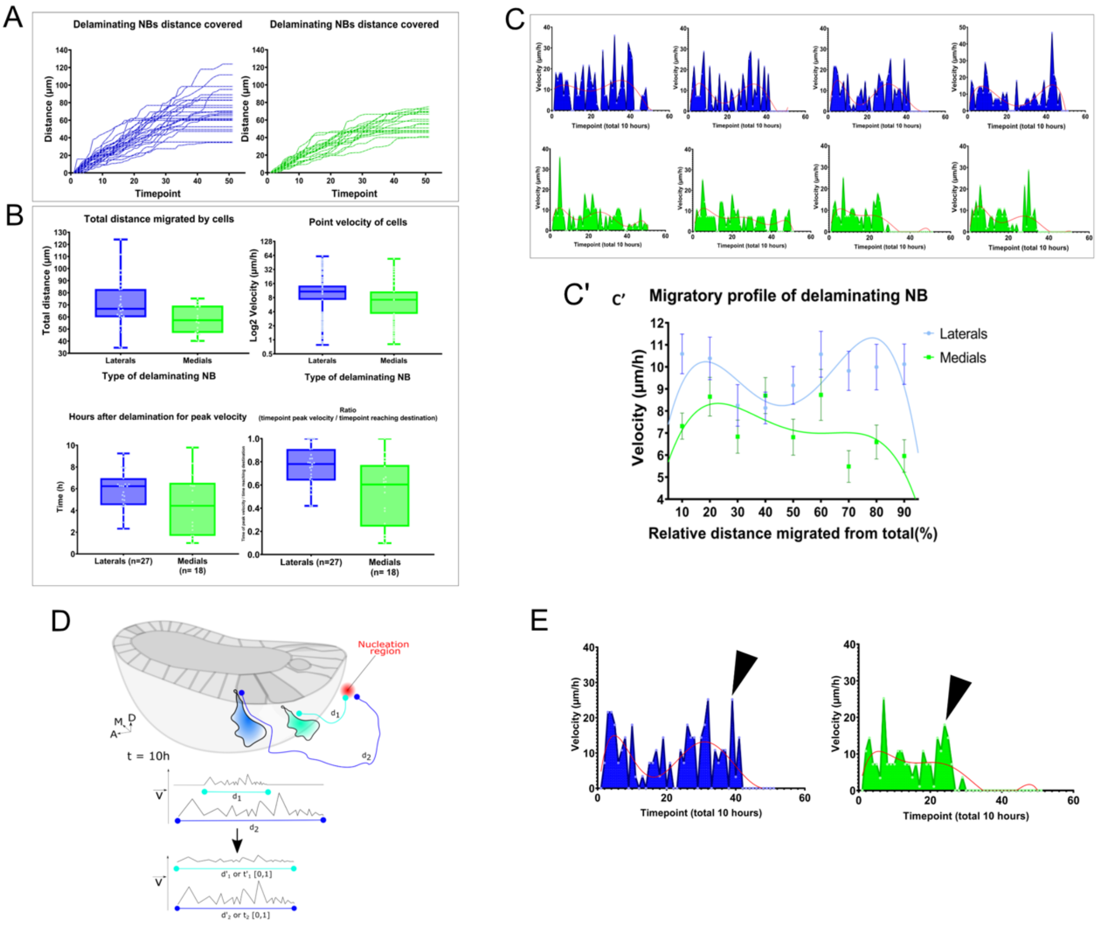

Fig. S8. Lateral versus medial NB migratory tracks, normalization and comparison. Migratory properties split between more lateral (blue, 27 cells) versus more ventromedial (green, 18 cells) delaminating NB (not all cases are included in this analysis). Graphics show from top to bottom and from left to right: A) distance covered of lateral, medial and merged NB ; B) boxplots of total migrated distance, velocity of cells, hours after delamination required for acquiring peak velocity and the ratio of “time at which NB acquire peak velocity / time at which NB reach destination”; C) eight graphics showing single cell migratory behaviors (4 lateral NB in blue and 4 medial NB in green) with a 6th polynomial spline fit for better visualization among velocity fluctuations; C’) a graphic showing common migratory behaviors of merged lateral (blue) and medial (green) delaminated NB. Whiskers show standard error of the mean (SEM). D) Graphic example of how velocity tracks are merged to get common properties to all migratory NB. Dark blue and light blue represent two delaminating NB that delaminate from different timepoints and from different positions. To be able to compare distinct migratory profiles, we normalized either distance or time. E) Example of two migratory profiles in which increased velocity while coalescing happens at different timepoints (black arrowhead). X axis is time (h), Y axis is velocity (µm/h). h: hours.

Development • Supplementary information

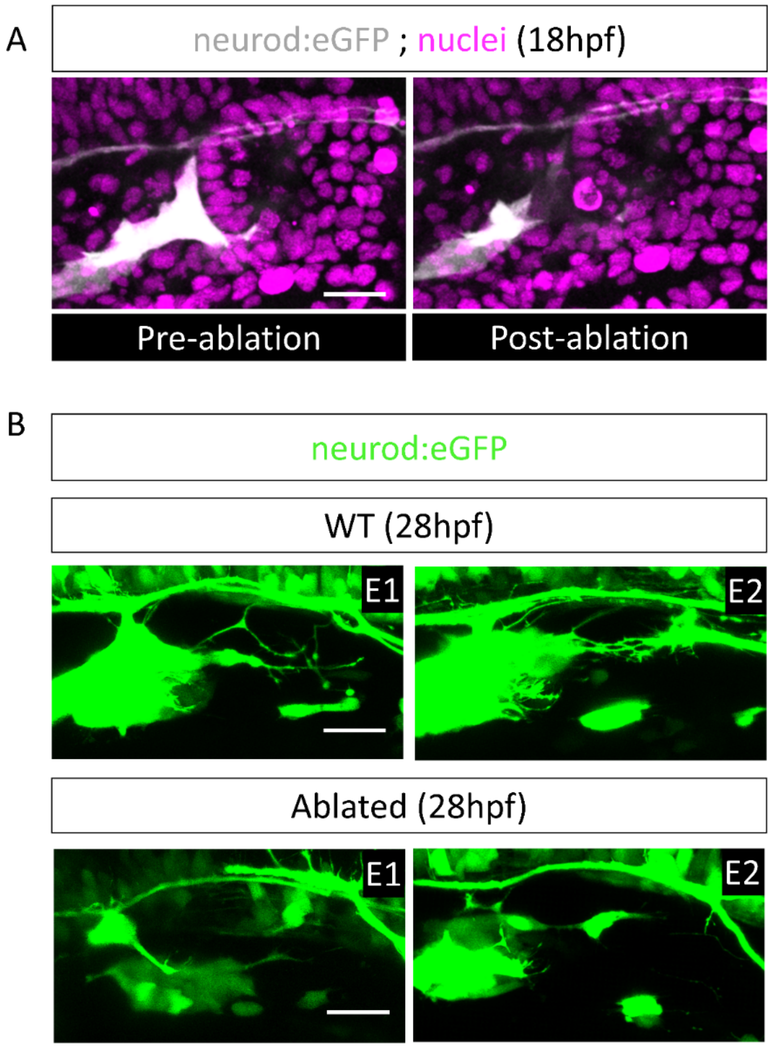

Fig. S9. "Late" pioneer SAG neurons ablation causes altered SAG shape. A) Ablating pioneer SAG neurons (*neurod* +) a little later (18hpf) in development (A) to avoid the possible effect of ablating also ingressing *neurogenin1*+ cells shows similar results to previously reported (B) at 28hpf, indicating again that ablating *neurod* population also causes SAG shape defects. Notice how only 4 to 5 cells are ablated in (A). Scale bar is 20µm.

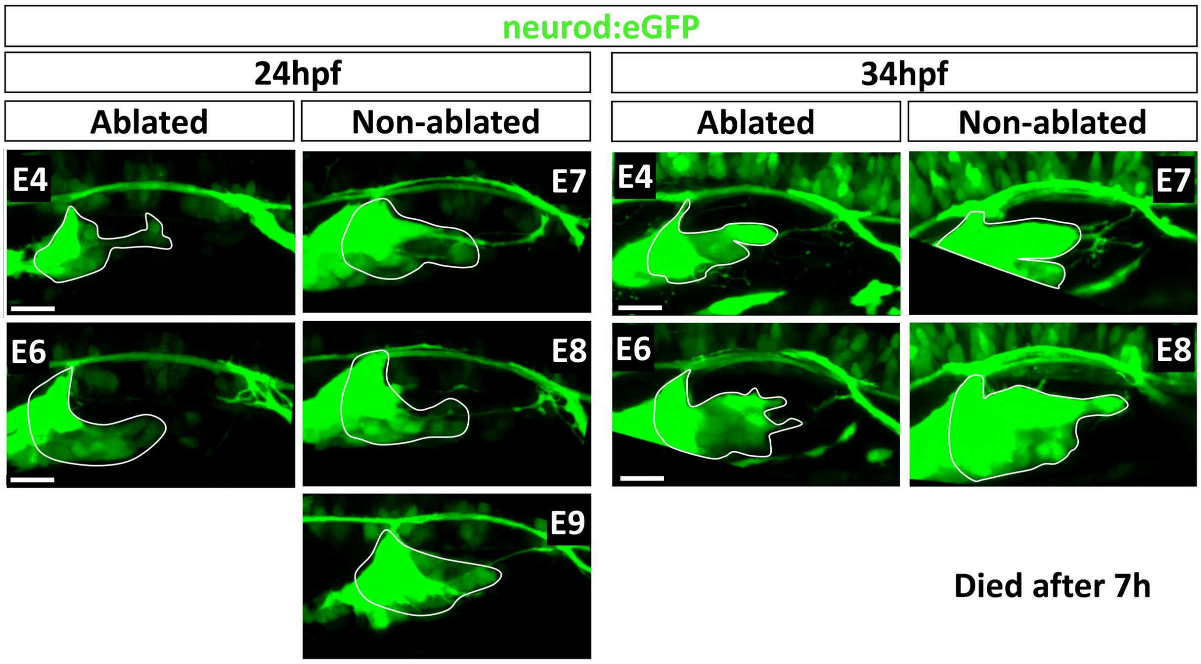

Fig. S10. Further examples of SAG shape in control versus pioneer SAG neurons ablated embryos. SAG shape is altered (white lines contouring eGFP signal) at both 24 and 34hpf. Also, there seems to be a reduced number of cells populating the SAG. Scale bar is 20µm. E: Embryo.

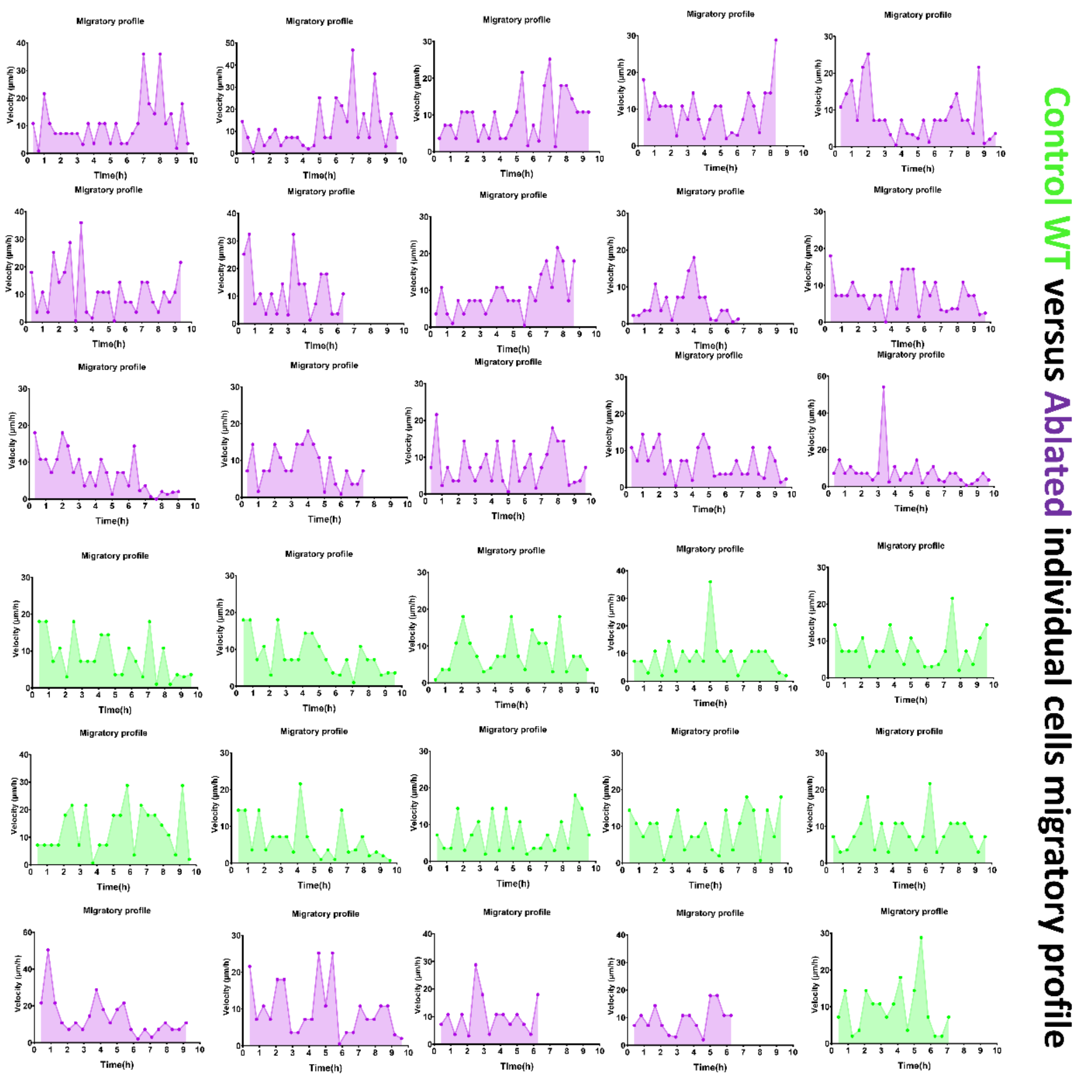

Fig. S11. Migratory profiles of single NB in ablated pioneer SAG neurons conditions versus controls. Migratory profile of single NB in control (green) or pioneer SAG neurons ablated condition (magenta). X axis, time (h). Y axis, velocity (µm/h). h: hours.

Development • Supplementary information

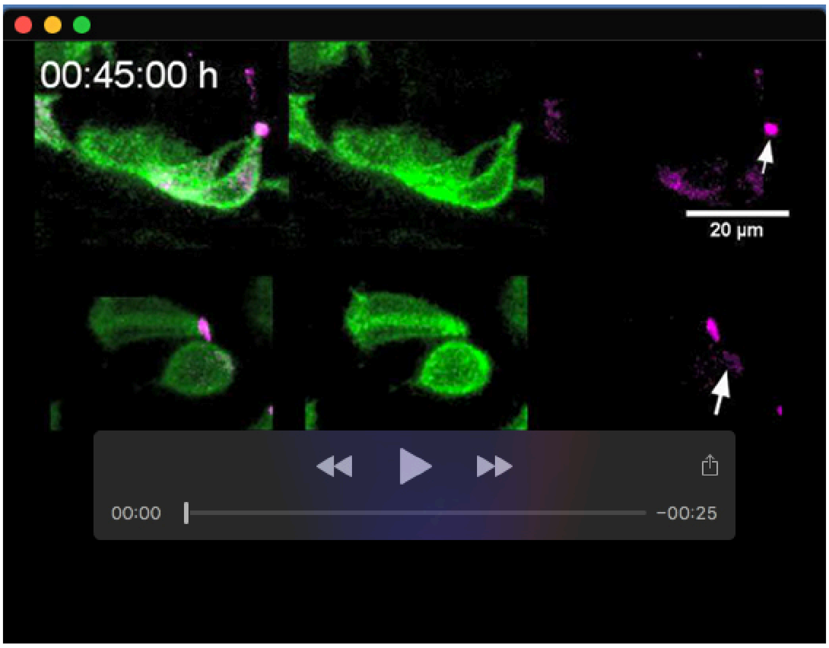

Movie 1. Delamination of a single-cell labeled otic NB. Time-lapse imaging of both a delaminating and a non-delaminating cell after injection at 32-64 cell stage embryo the mRNA for LynTdTomato (green) and Pard3-GFP (magenta). White arrows show pard3 puncta, yellow arrow shows apical thinning.

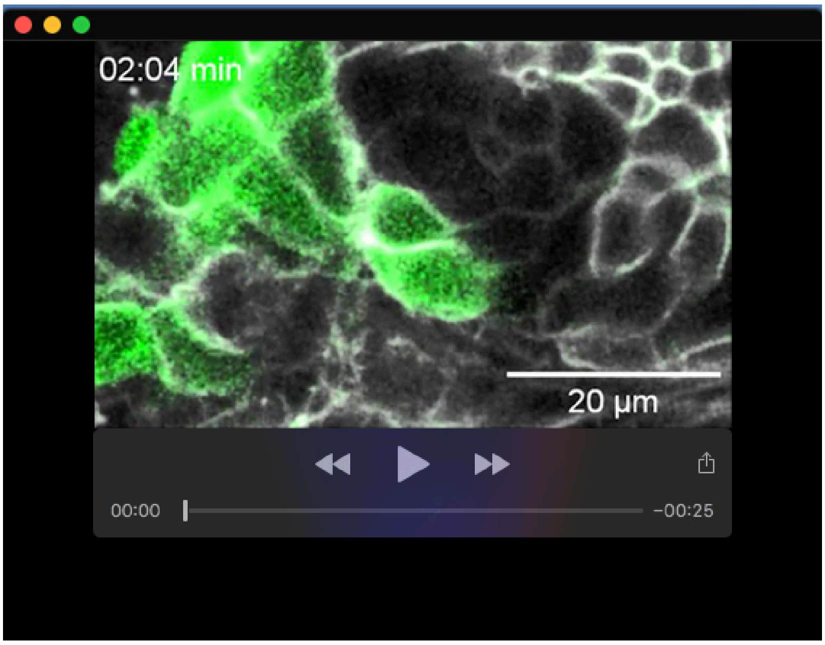

Movie 2. Blebs produced by an otic NB. Otic NB labeled with Tg(neurog1:dsRed; shown in green), membrane in white. White arrows indicate blebs.

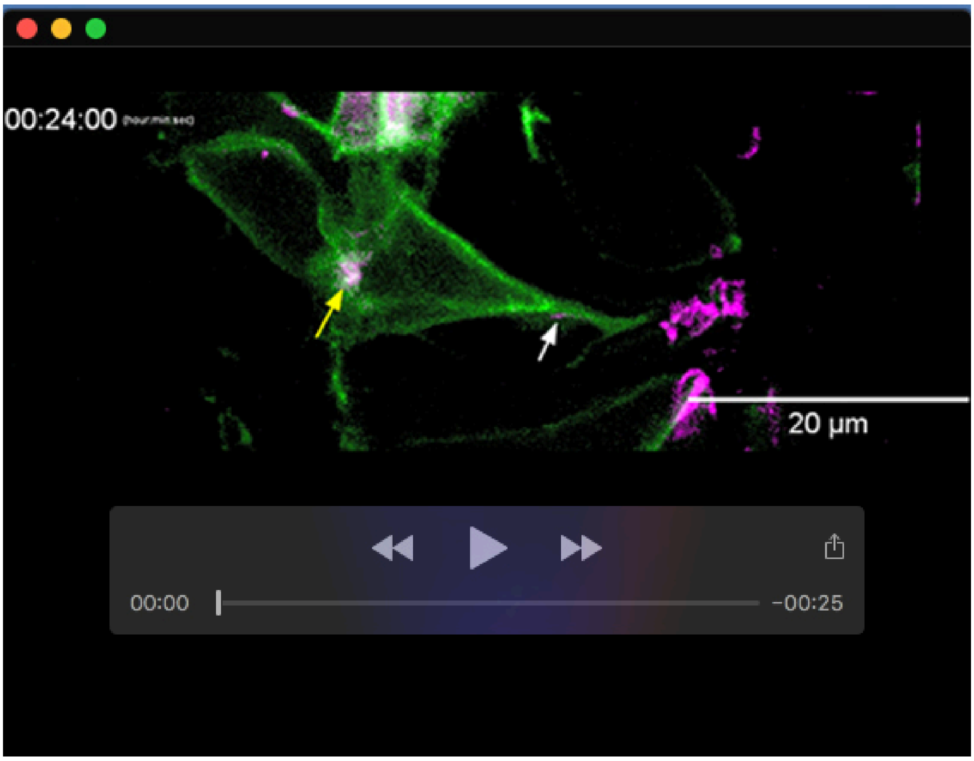

Movie 3. Pard3 dynamics in a delaminating NB. Pard3 (magenta) retrieved by the cell body of a delaminating NB (membrane in green) shown by white arrow. Pard3 basally located shown by yellow arrow.

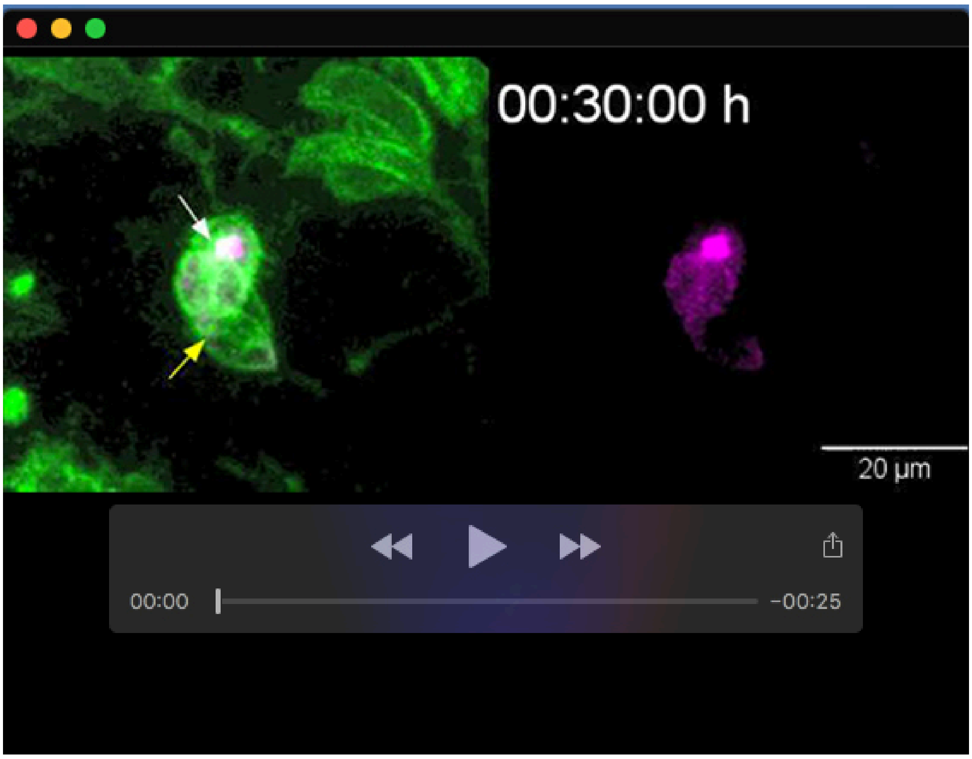

Movie 4. Pard3 dynamics in a group of already delaminated NB. Left panel: Delaminated NB separate with time (white versus yellow arrows). Right panel: Pard3 is dynamically redistributed in these cells.

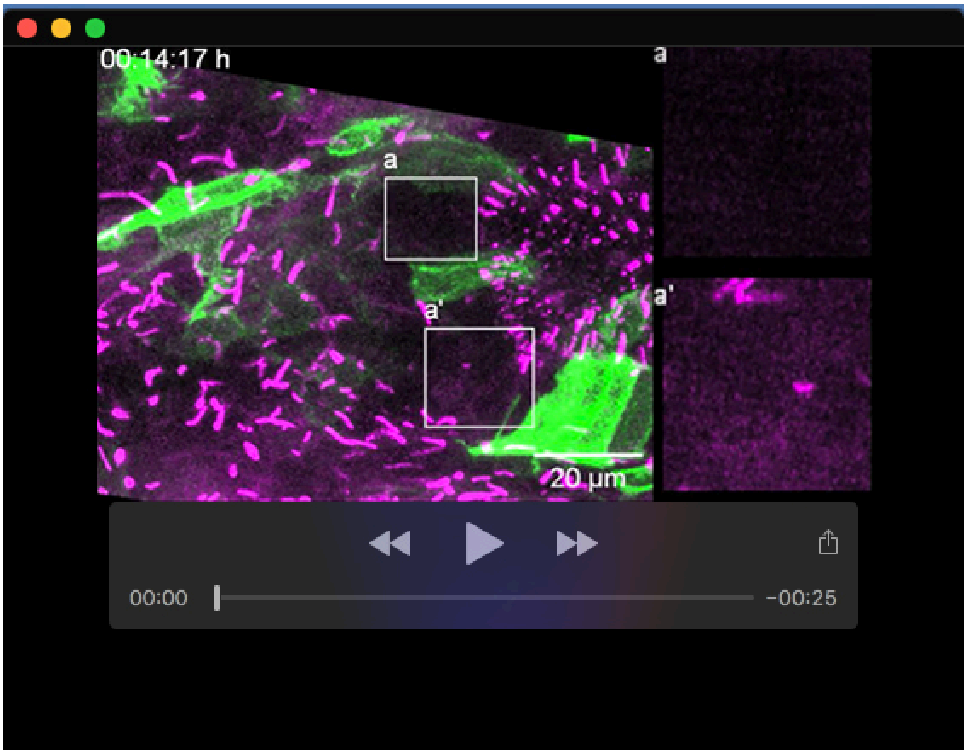

Movie 5. Arl13b dynamics in neurogenic (a') versus non-neurogenic (a) otic regions. Sparse membrane labeling in green, arl13b-GFP in magenta. Arl13b only goes through neuroepithelia in the neurogenic (a') region.

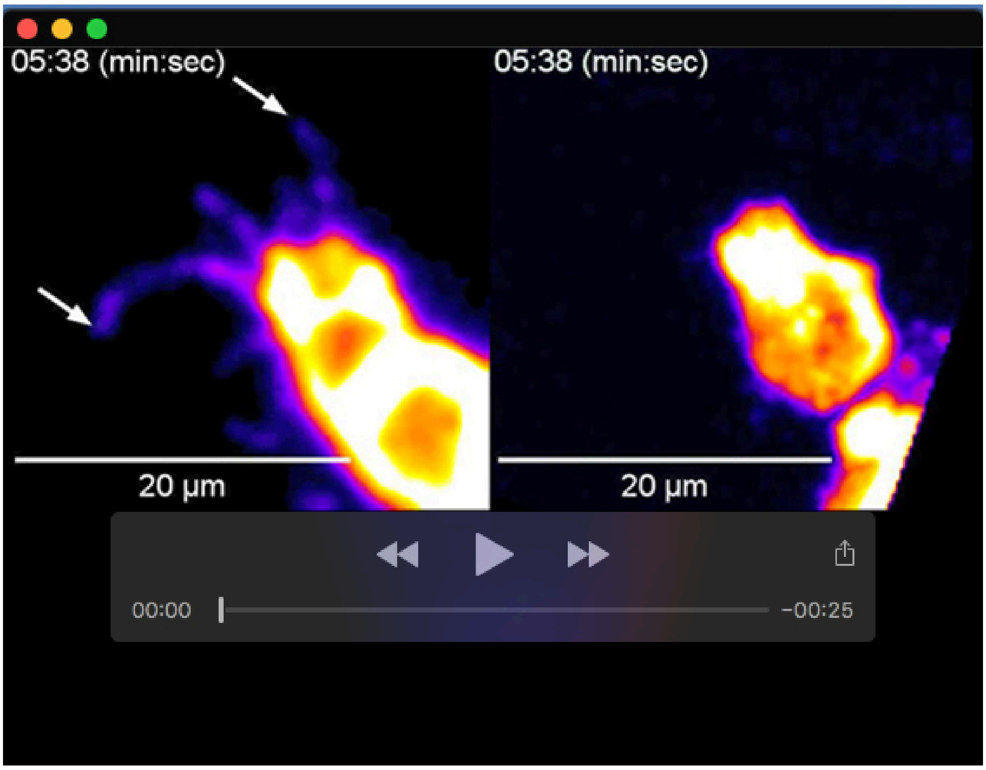

Movie 6. Filopodial processes in single cell membrane labeled delaminated otic NB. Membrane labeling by LynTdtomato shown with Fire LUT. White arrows show tips of filopodia. Green arrowhead shows bigger membrane deformation.

Development • Supplementary information

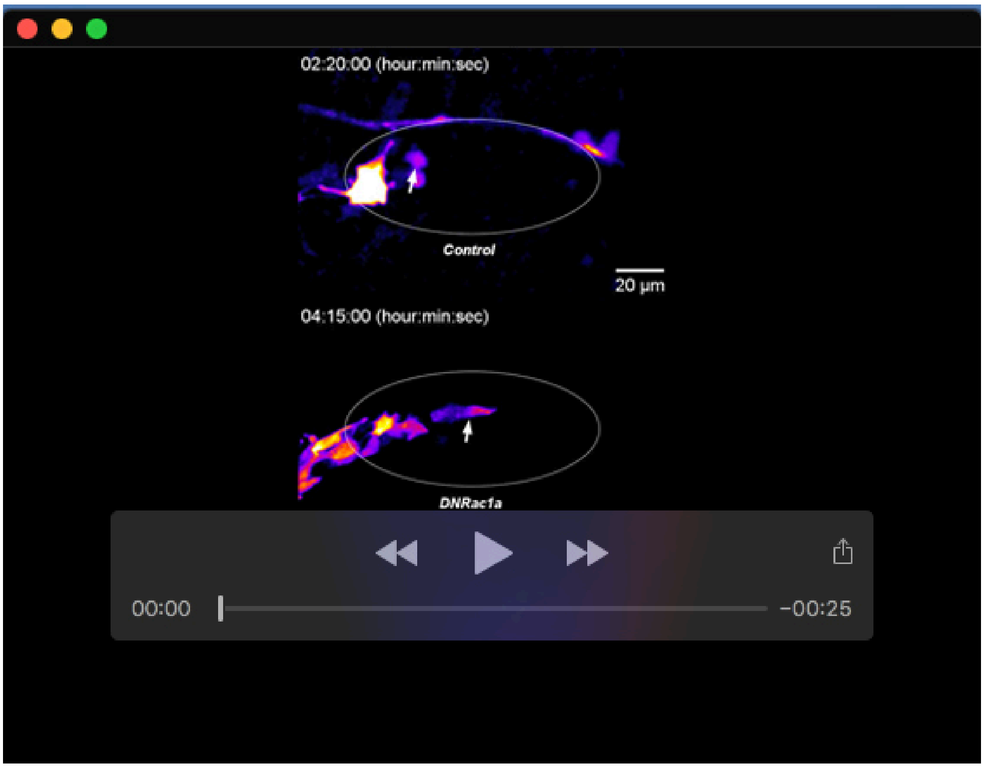

Movie 7. Migration of a delaminated otic NB in control conditions compared to a DNRac1a-expressing cell. Upper panel shows a control NB migration (white arrow). Lowe panel shows a DNRac1a-expressing NB migration(white arrow). Otic vesicle depicted with a white line ellipse.

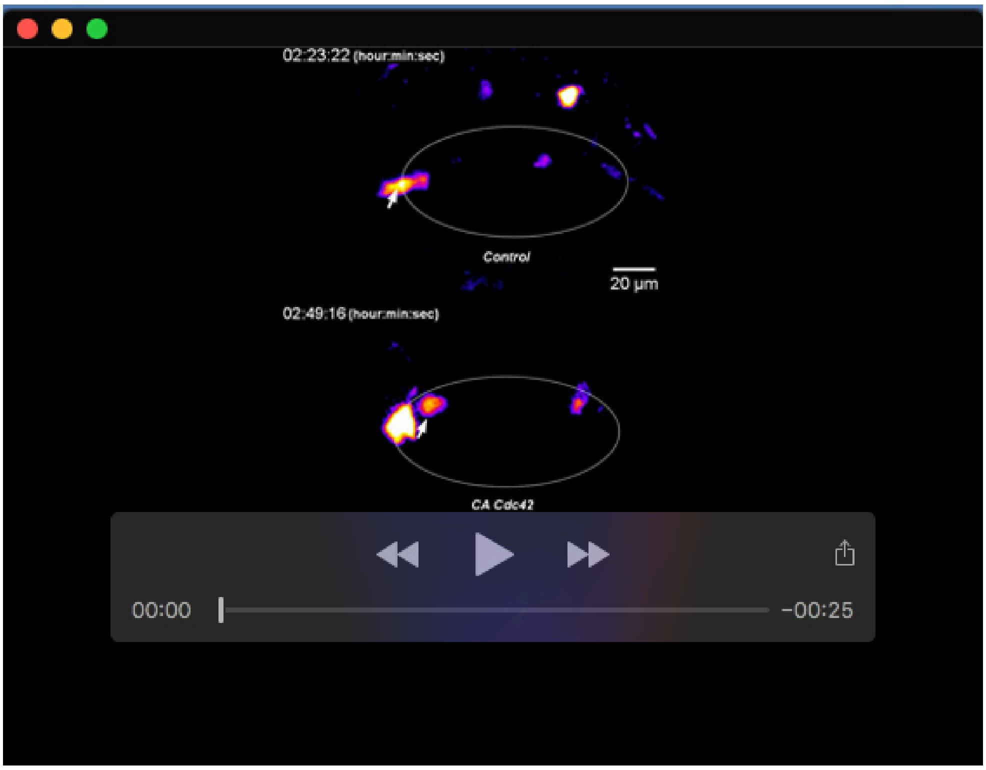

Movie 8. Migration of delaminated otic NB in control conditions compared to CACdc42-expressing cell. Upper panel shows a control NB migration (white arrow). Lowe panel shows a CACdc42-expressing NB migration(white arrow). Otic vesicle depicted with a white line ellipse.

Development • Supplementary information

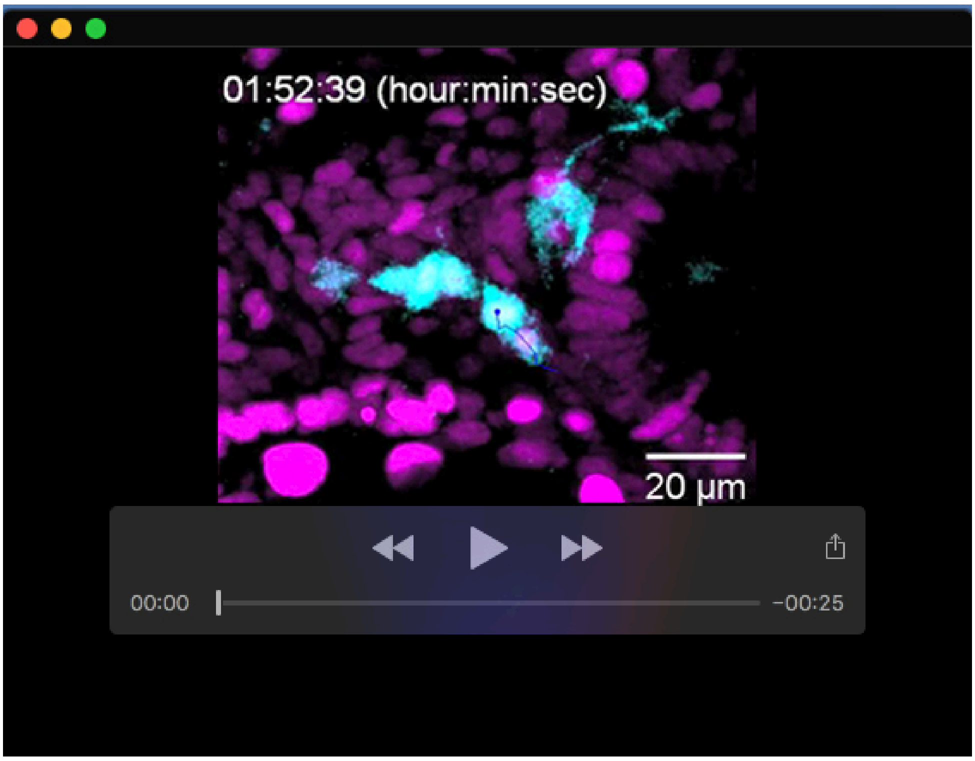

Movie 9. Migratory pathway followed by a delaminating NB. Pathway followed by a NB delaminating from the lateral neurogenic domain of the otic vesicle (blue track). The movie was made from the same raw confocal movie as Movie 11, but with different planes selected and contrast adjustments.

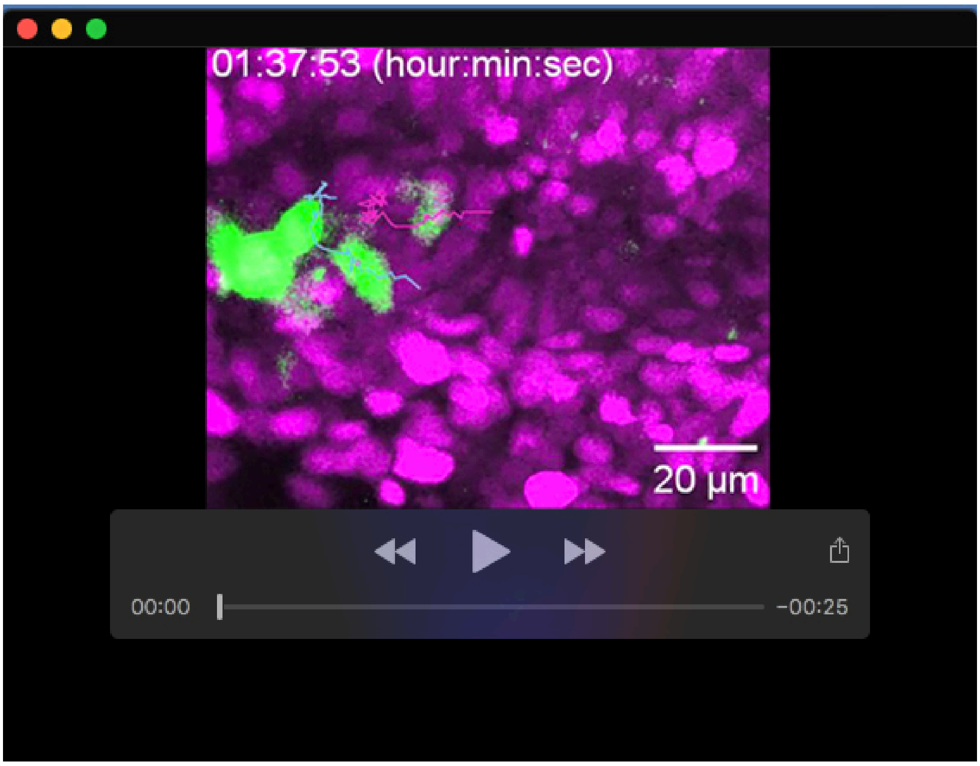

Movie 10. Migratory pathway followed by a delaminating NB. Pathway followed by two NB delaminating from the medial portion of the neurogenic domain of the otic vesicle (blue and magenta tracks).

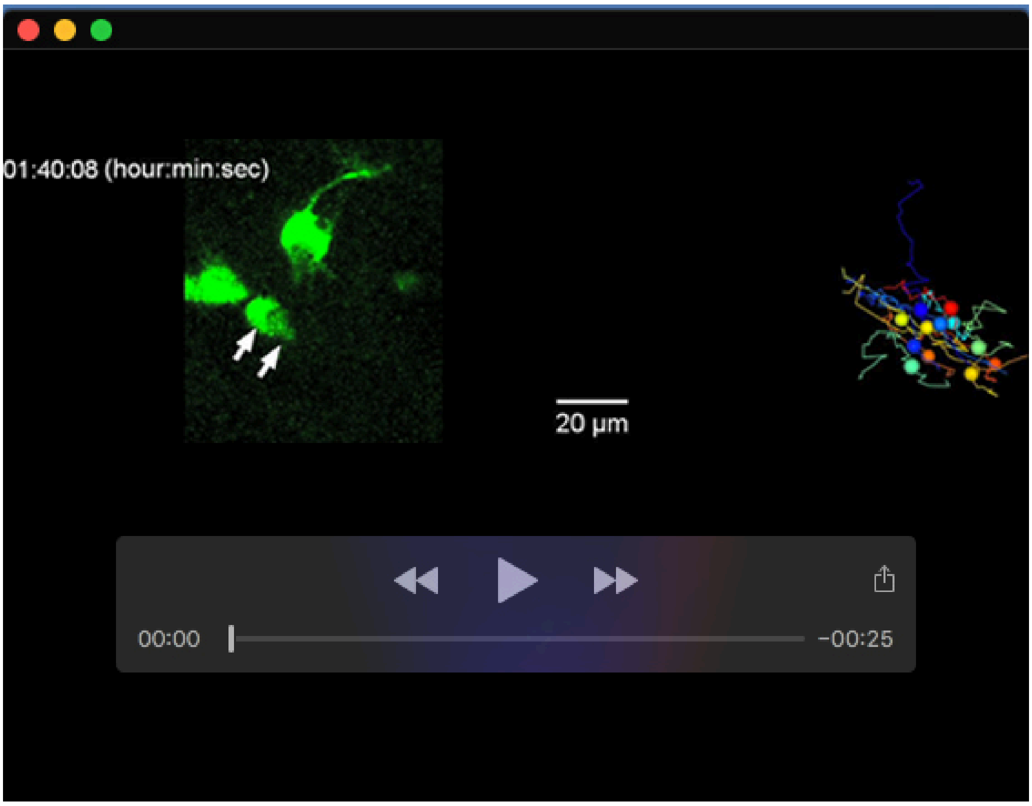

Movie 11. Non-collective migration of delaminated otic NB. Left panel shows NB (white arrows) separating with time. Right panel shows tracks of these NB and their neighbors. The movie was made from the same raw confocal movie as Movie 9, but with different places selected and contrast adjustments to visualize two contiguous cells.

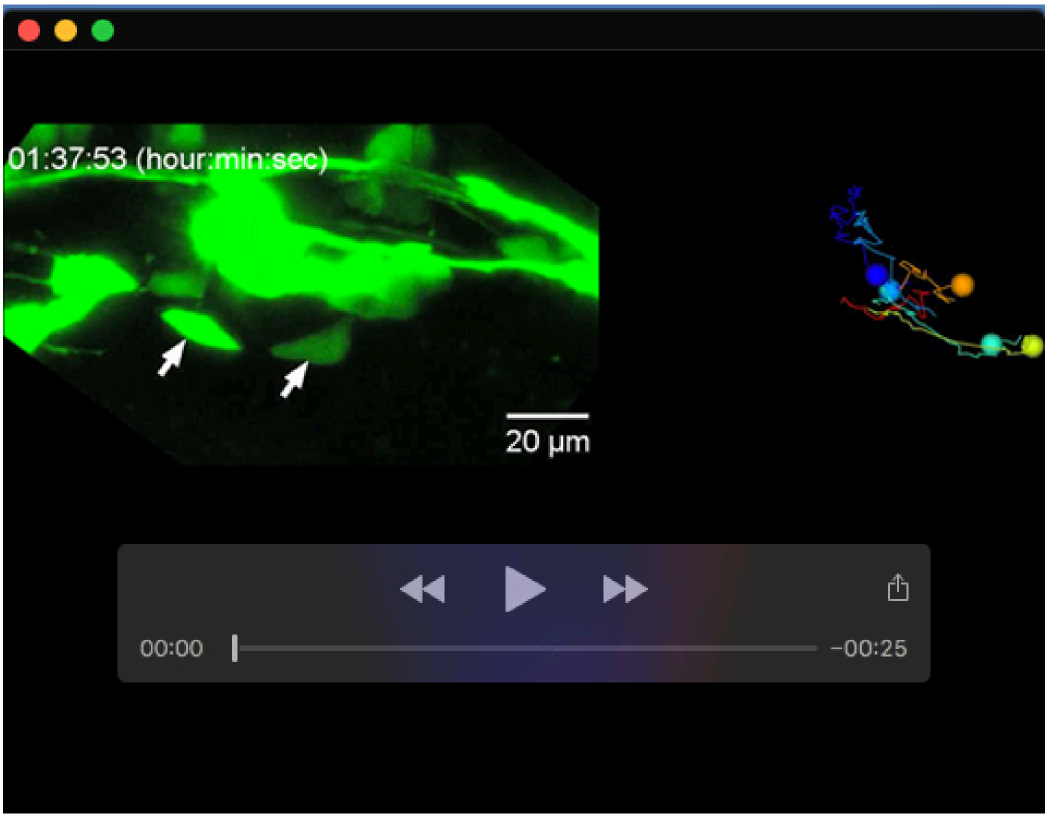

Movie 12. Collective migration of delaminated otic NB. Left panel shows two groups of NB (white arrows) staying together with time. Right panel shows tracks of these groups.

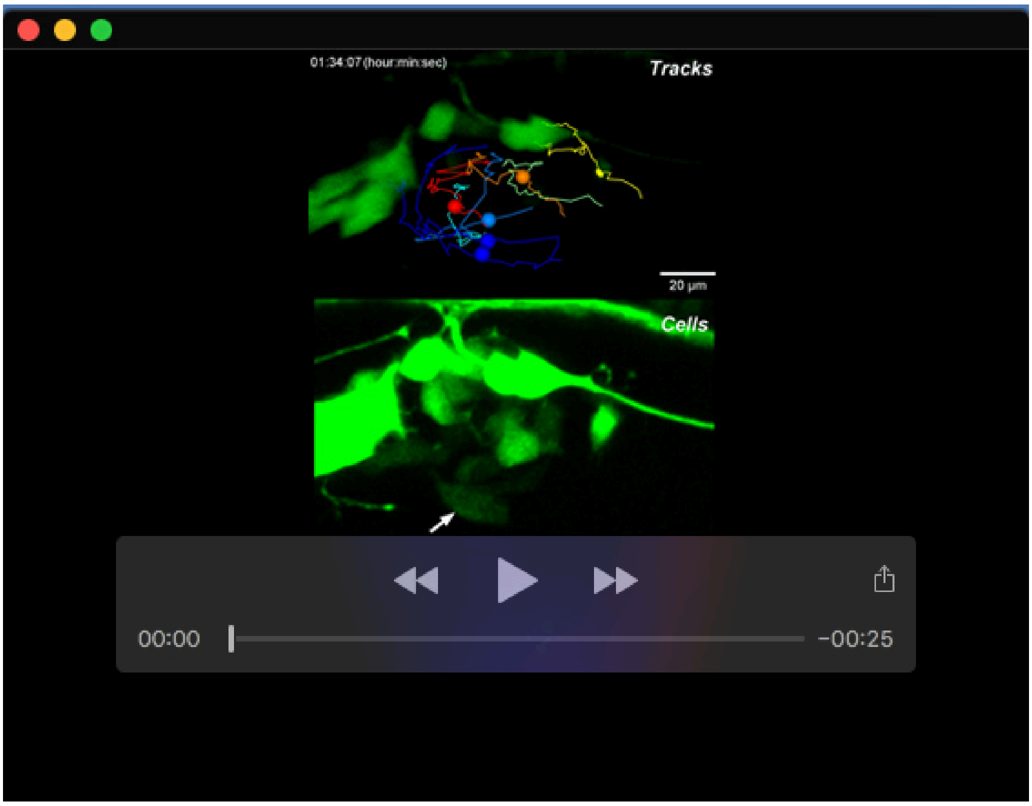

Movie 13. NB migratory pathways towards the coalescence region. Upper panel shows tracks of delaminated NB from lateral and medial positions of the neurogenic domain. Lower panel shows these NB (white arrows).

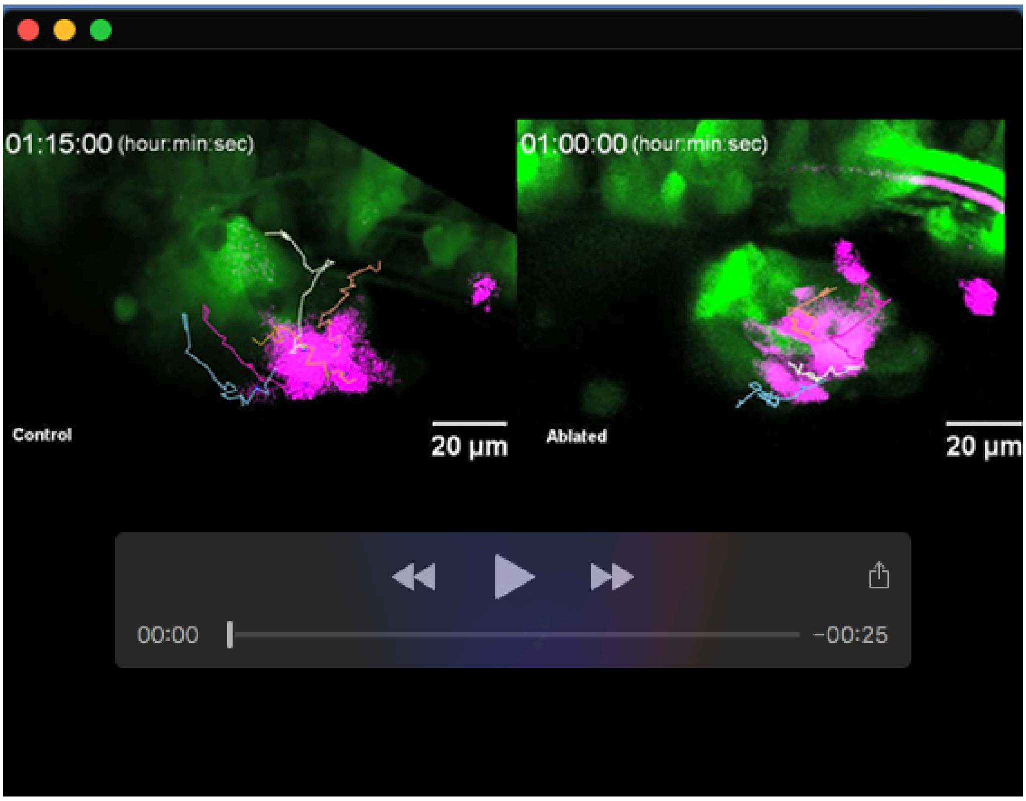

Movie 14. Migratory pathways followed by delaminated NB in control (left) and Pioneer SAG neurons ablated condition (right). After photoconversion of the same region of the neurogenic domain, NB in the ablated condition (right panel tracks) migrate aberrantly compared to control NB (left panel tracks).

Development • Supplementary information

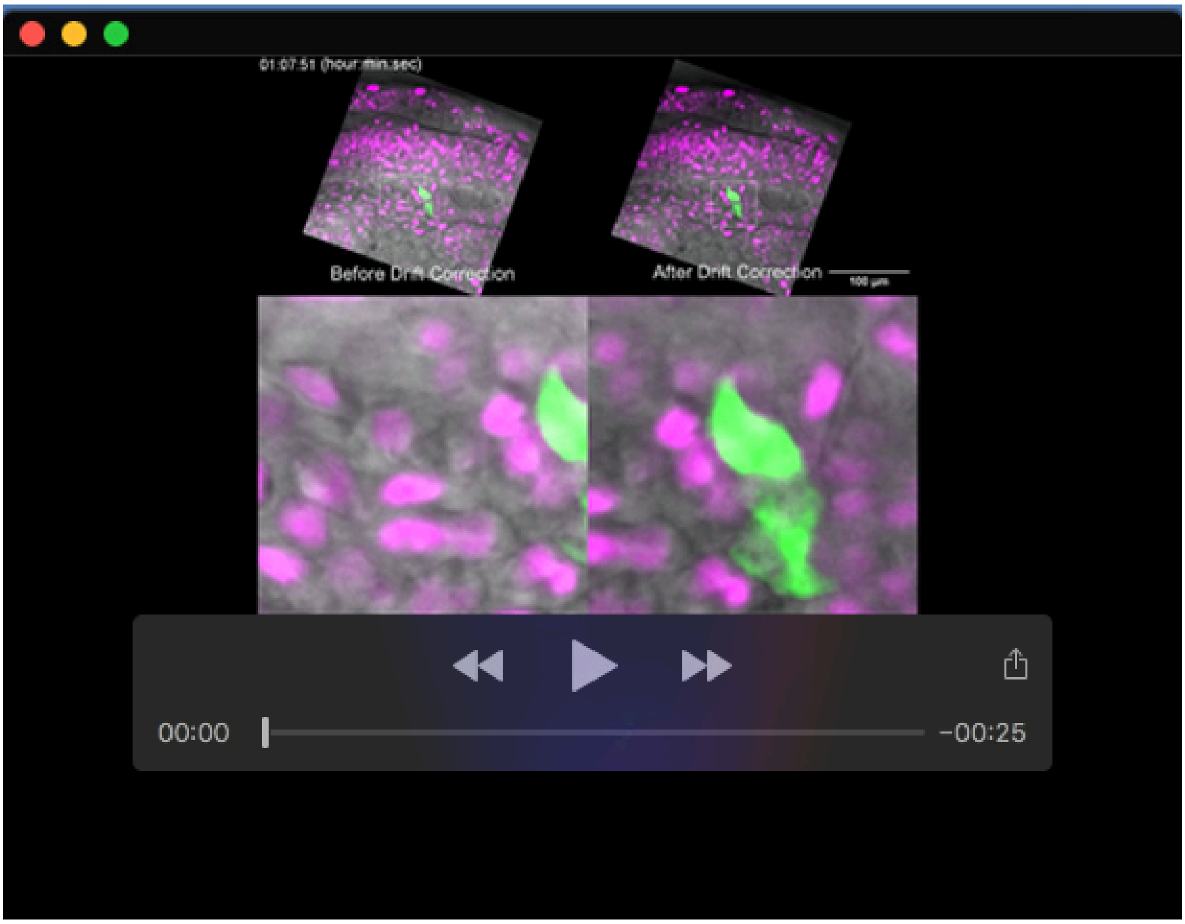

Movie 15. Morphogenetic drift correction of otic tissues. For drift correction, the otic vesicle is kept as a static reference. Therefore, NB migration exclusively can be addressed.
