# Supplementary material for: Pioneer statoacoustic neurons guide neuroblast behaviour during otic ganglion assembly
Source: Development. 2023 Nov 8;150(21):dev201824. doi: 10.1242/dev.201824 (PMC10651105; doi:10.1242/dev.201824)
Supplement: Supplementary file 1 [file develop-150-201824_review_history.pdf]

Pioneer statoacoustic neurons guide neuroblast behaviour during otic ganglion assembly

Aitor Bañón and Berta Alsina
DOI: 10.1242/dev.201824

Editor: Steve Wilson

Review timeline

Original submission:	30 March 2023
Editorial decision:	17 May 2023
First revision received:	27 July 2023
Editorial decision:	11 August 2023
Second revision received:	1 September 2023
Accepted:	7 September 2023

Original submission

First decision letter

MS ID#: DEVELOP/2023/201824

MS TITLE: Role of pioneer neurons and neuroblast behaviors on otic ganglion assembly

AUTHORS: Aitor Bañón and Berta Alsina

Apologies for the delay in obtaining reviews on your manuscript. One of the referees failed to return their report and so I have made a decision based upon the two reviews I have received. The referees' comments are appended below, or you can access them online: please go to BenchPress and click on the 'Manuscripts with Decisions' queue in the Author Area.

As you will see, the referees express considerable interest in your work, but have some criticisms and suggestions for improvements. Referee 2's comments almost all relate to writing style - I appreciate that we all have different writing preferences and it can be annoying to be told to conform to someone else's preferred style. However, clarity is critical and so please do consider whether this could be improved. If you are able to revise the manuscript along the lines suggested, I will be happy receive a revised version of the manuscript. Please also note that Development will normally permit only one round of major revision. If it would be helpful, you are welcome to contact us to discuss your revision in greater detail. Please send us a point-by-point response indicating your plans for addressing the referee's comments, and we will look over this and provide further guidance.

Please attend to all of the reviewers' comments and ensure that you clearly highlight all changes made in the revised manuscript. Please avoid using 'Tracked changes' in Word files as these are lost in PDF conversion. I should be grateful if you would also provide a point-by-point response detailing how you have dealt with the points raised by the reviewers in the 'Response to Reviewers' box. If you do not agree with any of their criticisms or suggestions please explain clearly why this is so.

© 2023. Published by The Company of Biologists under the terms of the Creative Commons Attribution License (https://creativecommons.org/licenses/by/4.0/).

Reviewer 1

Advance summary and potential significance to field

In this manuscript, Banon A and Alsina B describe how neuronal precursors of the stato-acoustic ganglion assemble into a cluster by tracking the delamination and migration neuroblasts in the zebrafish embryo. The authors found that neurobasts delaminate from otic epithelium through apical abscission and reorientation of the apical-basal axis of the cells. The delamination process involves formation of cellular filopodia and blebs, indicating an active cell migration machinery. Inhibiting or over-activating RhoGTPases (Rac, Cdc42, and Rho) changed the migration distance of neuroblast to their target, the site where pioneer statoacoustic ganglion (SAG) cells are located. Laser ablation of the pioneer SAG cells largely compromised the formation of ganglion. In general, the topic is of interest for the readership of the Development Journal, but issues related to the interpretation of polarity change in delamination and the RhoGTPase for delaminated neuromast migration should be addressed.

Comments for the author

1. “In addition, accumulation of Pard3 is also transiently detected in the basal cytoplasmatic domain in early delamination (Fig. 1E, yellow arrow in central panel), suggesting a possible change in polarity (Movie1 and 3)”. It is not clear how Par3 is distributed in cells that do not delaminate. Therefore, it is not known whether the Par3 re-distribution during delamination is caused by the change of cellular polarity. It would be interesting to observe how Par3 starts to appear on the basal sides of the neuroblasts before or during the delamination. One would expect that control cells do not show such a re-distribution.

2. “This suggests that in spite of a mesenchymal phenotype, polarity is reorganized and could be the driving force for NB dispersion (Fig. 1G and Movie 4)”. This could be tested by directly inhibiting par3 or other apical determinants in the neuroblasts and checking whether delamination still happens.

3. Figure 1H. It would be helpful if the membrane was also labeled as was done for the Par3 panels.

4. “Once delaminated, NB modify their roundness, extend filopodial protrusions that later on translate into larger membrane deformations”. The authors do not show this. Filopodia and protrusions could form independently of each other. Either the authors show that this is indeed the case or they should modify their statement.

5. “Delaminating NB suffer a process of apical thinning and abscission but Pard3 and Arl13b components are not lost and relocate in the delaminated cell, suggesting that can help establish a new polarity front in NB”. What’s the function of the relocation of Pard3 and Arl13b? Are they related to the later directional migration of the cells?

6. “This indicates that the directionality of migration in DN-expressing NB is impaired.” The directionality of migration in DNRac1-expressing NB can be directly measured as the author did in Figure 4C.

7. “We extended the analysis by abrogating (DN) and increasing (CA) the function of the RhoGTPases Cdc42 and Rho1 in otic NB (Fig. 3B-C, Movie 8). In these conditions, we found that NB have a reduced or recovered migratory capacities, respectively (Fig. 3C, asterisk)”. There is no quantification of the data for the conditions in which Cdc42 and Rho1 are abrogated or increased. Also, it is unclear what the authors mean by “recovered migratory capacity – does caCdc42 or caRho1 not impair migration or increase motility?

8. If RhoGTPases regulate the migration of delaminated neuroblasts, what are the consequences of deregulating RhoGTPase for the final formation of SAG?

9. Additional comments

- Line 157, “Fig. 2C” should be “Fig. 1G”

© 2023. Published by The Company of Biologists under the terms of the Creative Commons Attribution License (https://creativecommons.org/licenses/by/4.0/).

- Line 360, “Fig. 6C’-C’’” is not labelled in the main figure.
- The authors need someone to correct the grammar and wording inaccuracies in the paper.

Reviewer 2

Advance summary and potential significance to field

Bañón and Alsina report their findings while studying the dynamics of SAG morphogenesis in zebrafish. They conclude that non-otic pioneer neurons are essential to guide ganglion shape development. The work uses a variety of relevant and novel tools, generating an impressive high-quality dataset. Their main finding is important and will be interesting for scientists within and beyond the immediate field. The CRISPR Eraser is a cool tool. Very nicely employed.

Comments for the author

My main concerns are about form, text and assumptions. First, the tile is not terribly informative. They should consider something along the lines of "Pioneer neurons guide neuroblast behavior during otic ganglion assembly"

There are (too) many sentences that are oddly written, or written in a passive voice, which makes the text tiresome. Below a selected set of examples

1. It is little understood how individual neurons coalesce, distribute and shape the ganglion.
2. "... imaging and analysis of individual neuronal dynamics during SAG development has poorly been addressed"
3. "In chick and zebrafish, it has been shown that the most anterolateral portion of the neurogenic domain generates vestibular neurons..." (delete: it has been shown that).
4. "...has revealed novel information on cell shape changes and behaviours during cell delamination, division or migration" (I do not understand this sentence).
5. "Radial glial cell migration in the cortical plate is a long-known mechanism by which newborn neurons use progenitor cell projections to properly migrate at later stages"(I find this incomprehensible).
6. "Specially studied in invertebrates is the modulation of neuronal dynamics during development by pioneer cells." (what is "Specially studied"?).
7. "However, other evidences suggest the contrary, where published gene expression data and our imaging data points towards a delamination process in the inner ear with EMT characteristics." Please rephrase.
8. "...some membrane remaining’s," (what is membrane remaining’s?)

"Thus, for a correct circuitry between hair cells and otic neurons, SAG morphogenesis must be tightly coordinated in time and space with otic tissue development." Needs a reference.

"...consists of an oval epithelium with an internal..." (the otic placode is not an oval epithelium, but and epithelial oval)

In "During this cell shape rearrangement, the apical domain becomes narrower in a zipper-like process, to finally become a thin membranous filament (arrowheads in Fig. 1C)."
Does it make sense to call "apical" a domain in a mesenchymal cell? Especially because from their Par3 data it may be that the observed cell has either two apices or none. Also, they state that "experiments and literature suggest that otic NB undergo an EMT process when delaminating."

"unpublished results from the laboratory" Show or don't mention them.

The kymographs in Fig.1H’ and H’’ are difficult to understand

"Comparing quantified velocities across time (Fig. 3G left graph and Supplementary figure 4)" "across time" can be deleted

© 2023. Published by The Company of Biologists under the terms of the Creative Commons Attribution License (https://creativecommons.org/licenses/by/4.0/).

I do not understand what they are trying to say in the following paragraph
"We have also seen NB can migrate either collectively or non-collectively. In a collective migration, cells can move cryptically within the cluster (Reig et al., 2014) but the overall migration is robust. In NCC tissue, E-cadherin helps maintaining stream migration while Pard3-N-Cadherin interactions avoids crowding by contact-inhibition of locomotion (Clay & Halloran, 2014; Moore et al., 2013; Priya & Yap, 2015; Scarpa et al., 2015). If a temporarily regulated and partial switch in adhesion molecules is necessary to maintain some sort of collective migration for keeping tissue cohesiveness remains to be studied. We do know that itg5a is required for correct aggregation of epibranchial placodes (Bhat & Riley 2011), maintaining tissue segregation from other placodes and cohesiveness within the tissue."

"Using in vivo resonant scanning for higher temporal resolution..."
This is not correct. Resonant scanning imaging perhaps? In any case, this an other technical details do not need to go in the main text. They seem to have been placed here to create a "wow effect", which in my opinion is not necessary.

Delete "." in line 140

First revision

Author response to reviewers' comments

We appreciate the comments from the reviewers. We have addressed point by point the reviewers' concerns.

Regarding reviewer 1:

1.- *"In addition, accumulation of Pard3 is also transiently detected in the basal cytoplasmatic domain in early delamination (Fig. 1E, yellow arrow in central panel), suggesting a possible change in polarity (Movie1 and 3)". It is not clear how Par3 is distributed in cells that do not delaminate. Therefore, it is not known whether the Par3 re-distribution during delamination is caused by the change of cellular polarity. It would be interesting to observe how Par3 starts to appear on the basal sides of the neuroblasts before or during the delamination. One would expect that control cells do not show such a re-distribution.*

We appreciate the comment and in trying to answer it, we have assembled and included a new supplementary figure and improved **Movie1** to show Pard3 dynamics in both delaminating and non-delaminating regions. In the latter, Pard3 is apically localized for the whole span of the recording and does not change its position, in contraposition with Pard3 in delaminating cells, which change location. Moreover, In the new supplementary figure (which now is **Sup. Fig.1**) we also show how Pard3 starts to appear on basal sides of NB before delamination.

© 2023. Published by The Company of Biologists under the terms of the Creative Commons Attribution License (https://creativecommons.org/licenses/by/4.0/).

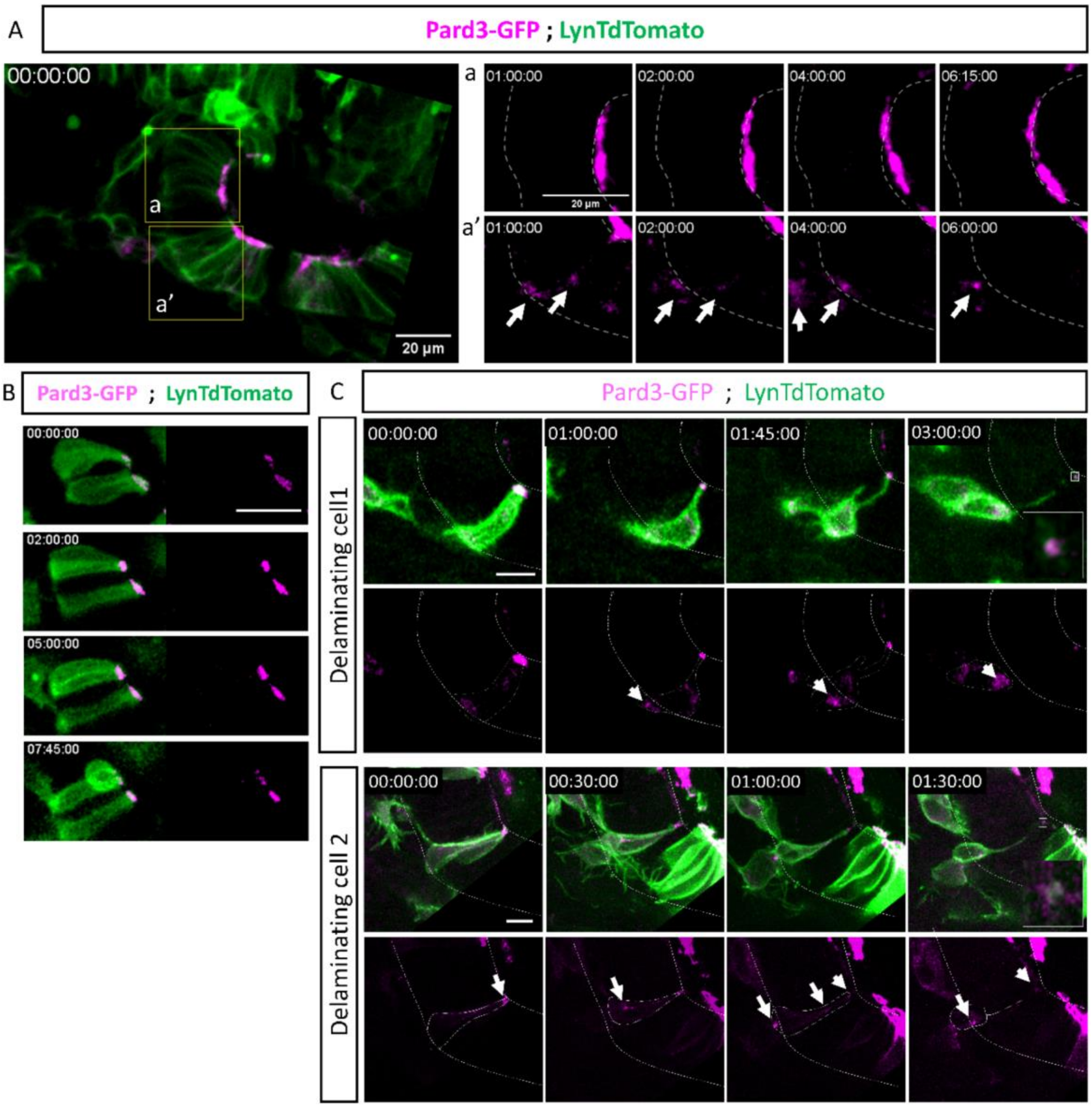

Supplementary Figure1. Pard3 dynamics in non-delaminating and delaminating cells. A) Pard3 remains apical in non- delaminating otic regions (a) while it is relocated to basal side in delaminating cells (white arrowhead in a'). B) Membrane (LynTdTomato, green) and Pard3 labelling (magenta) in non-delaminating otic cells showing how these cells do not change their columnar shape and Pard3 is not relocated to basal sides (apical side to the right). C) Pard3 relocation (white arrows) in two more examples of delaminating NB (depicted with white dashed line) before and during delamination. Apical always to the right. Scale bar is 20μm when noted, otherwise is 10μm.

2- *"This suggests that in spite of a mesenchymal phenotype, polarity is reorganized and could bethe driving force for NB dispersion (Fig. 1G and Movie 4)". This could be tested by directly inhibiting par3 or other apical determinants in the neuroblasts and checking whether delamination still happens.*

We are aware that this would be the best approach. However, with the tools we have in the lab it would be very difficult to address this question precisely. We do not have a UAS-DNPard3 nor an Opto-Pard3 to be photoactivated. Moreover, UAS activation using neurod: Gal4 line is mostly in delaminated cells and activating it inside the neuroepithelium is quite inefficient. Moreover, the use of morpholinos or CRISPR/Cas9 would affect embryo development and thus draw proper conclusions could be obscured due to broad developmental abnormalities.

To approach the question, we performed the following experiments, which have been included as **Sup. Fig. 2.** The experiments consisted on 1) addressing force dynamics through a reporter of myosin (*Tg(actb2:myl12.1-mCherry)*) under Pard3-mRNA injection and tracking in vivo; and 2) force inhibition by UAS-DNRac1a together with Pard3 mRNA injection.

© 2023. Published by The Company of Biologists under the terms of the Creative Commons Attribution License (https://creativecommons.org/licenses/by/4.0/).

The results show that while Pard3 relocates, myosin II enhanced signal follows that of Pard3. The correlation between Pard3 and contraction, suggest a possible contribution of Pard3 in generating forces when NB delaminate. However, it is possible that contraction by myosin II mediates Pard3 redistribution.
Pard3 relocates under DNRac1a conditions, however NB fail to delaminate, suggesting delamination is an active process mediated by RhoGTPases.
In any case, we thank the reviewer for the comment, and we toned down the sentence as it could have been an overstatement.

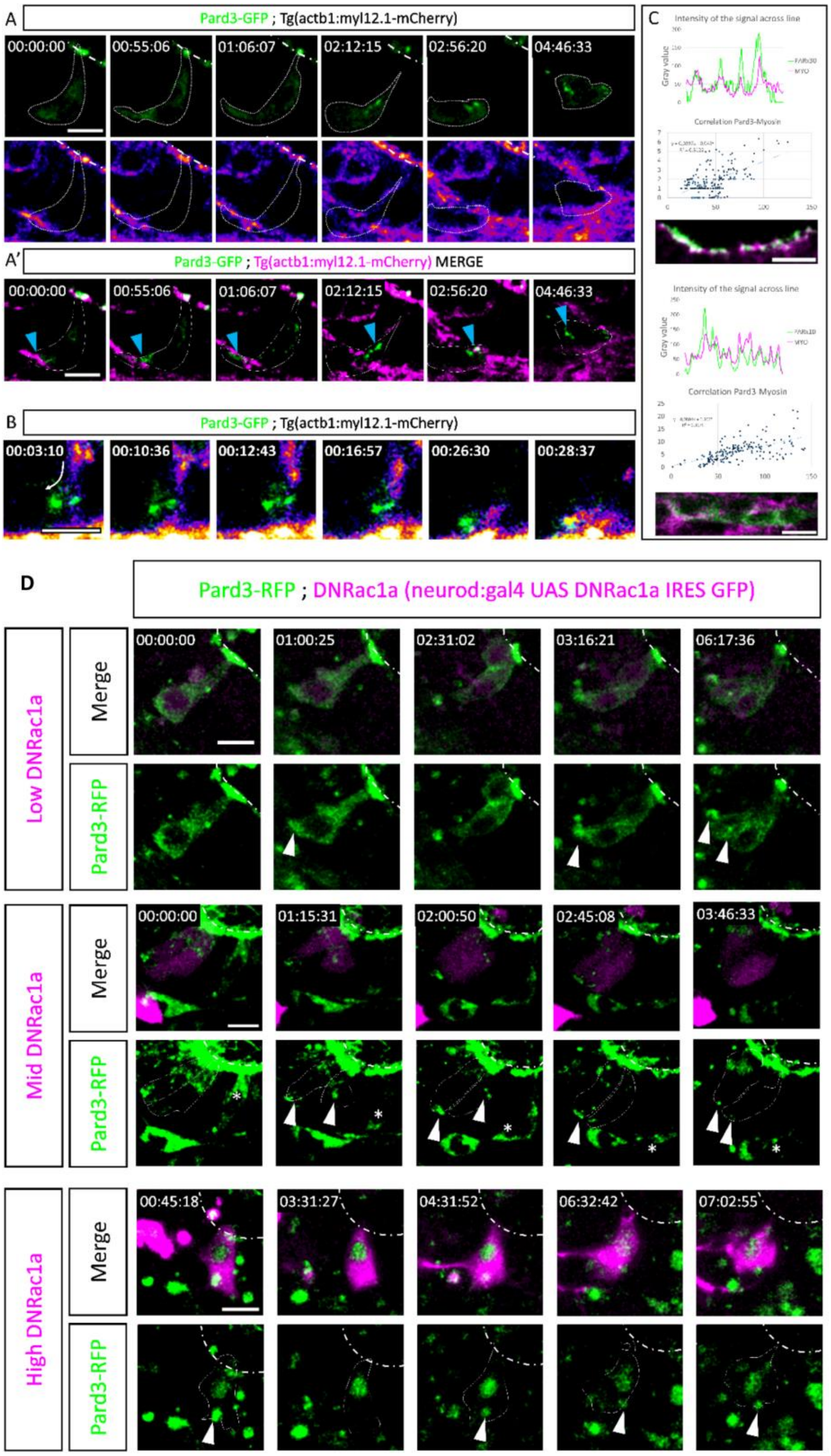

© 2023. Published by The Company of Biologists under the terms of the Creative Commons Attribution License (https://creativecommons.org/licenses/by/4.0/).

Supplementary figure 2. Pard3 and myosin dynamics. A) Pard3 (green) relocates as previously described. Myosin enhanced signal (Fire LUT, 8-bit gray scale value 255 is yellow, while close to 0 is purple/black) is found in Pard3 vicinities, which can be appreciated together in panel A' (blue arrowheads). B) Higher temporal and spatial resolution experiment shows pard3 and myosin of a delaminating NB. Myosin apparently follows Pard3 from behind. C) Co- localisation analysis of Pard3 and myosin in apical domains (upper panels) and after delamination (lower panels), indicating that increased pard3 signal relates to increased myosin signal in pard3 vicinities. D) Rac1a RhoGTPase inhibition (DNRac1a, magenta) shows pard3 relocates (green, white arrowheads) to basal side of the cell. However, NB fail to delaminate. A delaminating NB not expressing DNRac1a (middle panel, asterisk [*]) shows pard3 relocation and delamination. Cells are outlined in white dashed line. Apical side depicted by white dashed and dotted line. Scale bars are 10μm.

3- *Figure 1H. It would be helpful if the membrane was also labeled as was done for the Par3 panels.*
We have substituted panel H from Fig. 1 for a new panel showing the membrane of the otic vesicle labelled.

4-. "Once delaminated, NB modify their roundness, extend filopodial protrusions that later on translate into larger membrane deformations". The authors do not show this. Filopodia and protrusions could form independently of each other. Either the authors show that this is indeed the case or they should modify their statement.

It is true that the sentence implied a temporal sequence. We have modified the statement for: "Once delaminated, NB modify their roundness, extend filopodial protrusions and larger membrane deformations"

.5- "Delaminating NB suffer a process of apical thinning and abscission but Pard3 and Arl13b components are not lost and relocate in the delaminated cell, suggesting that can help establish a new polarity front in NB". What's the function of the relocation of Pard3 and Arl13b? Are they related to the later directional migration of the cells?
The apical protein Pard3 is required for neural crest migration (Moore et al., 2013). Pard3 also acts as an apical organizer (Afonso & Henrique, 2006) and apicobasal polarity is compromised in delaminating cells from the neural tube in chick (Das & Storey, 2014; Kasioulis et al., 2017). Burute et al., 2017 show in an *in vitro* model of mammary gland development that delamination of cells undergoing EMT is mediated by a reversal of cell polarity, in which the centrosome (from which Arl13b could be related and retained in our case) repositions at the cell center by a partial disassembly of the microtubule network and the release of Par3 from intercellular apical junctions. In our case we describe otic delaminating NB undergo Pard3 and Arl13b changes while delaminating. Relocating these components might be relevant for later organization of the microtubule mesh and forces for cell migration upon RhoGTPases.

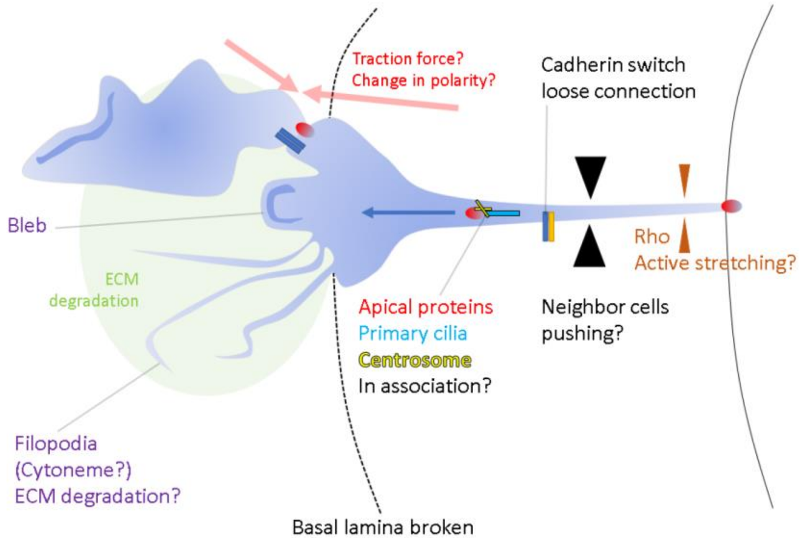

© 2023. Published by The Company of Biologists under the terms of the Creative Commons Attribution License (https://creativecommons.org/licenses/by/4.0/).

Directed migration is reflected by reorganization of microtubules, actin machinery and the centrosome. Active Cdc42 and Rac1 target apical polarity complex proteins Par3/6 and aPKC, which makes the microtubule organizing center (MTOC) and Golgi apparatus to locate in front of the nucleus (Raftopoulou & Hall, 2004; Reig et al., 2014; Ridley et al., 2003) and engage actin machinery to enhance its activity, promoting front-rear polarity.
It has been reported that apical polarity protein Pard3 controls NCC migration (Moore et al., 2013) promoting microtubule catastrophe against Rac1a-mediated microtubule stabilization. Thus, Pard3 presence prevents cells from migrating in the same direction. Findings from Burute et al., 2017 show loss of apical Pard3 leading microtubule destabilization regulates centrosome repositioning to cell center, and centrosome repositioning favors cell scattering. This lines up with complementary findings from Hong et al., 2010 in which Pard3 apical accumulation relocates the centrosome to apical positions in mesenchymal-to-epithelial transition (MET). Consequently, we can argue that there are cases in which Pard3 controls or interacts with centrosome positioning, modulating directed migration.
6. *"This indicates that the directionality of migration in DN-expressing NB is impaired." The directionality of migration in DNRac1-expressing NB can be directly measured as the author did in Figure 4C.*
The word "directionality was incorrectly used. We meant "directed migratory pathway" in contraposition to random walk or more recurrent shifting in the direction of migration. We changed the word "directionality" for "consistent migratory direction/pathway". Moreover, we did not include the polar plots in this condition since polar plots for DN are not as informative. Although less directed, the net vector resembles to that of WT. Thus, the complete track is more informative. Nevertheless, we have included them in the **Sup. Fig. 6**) as shown below:

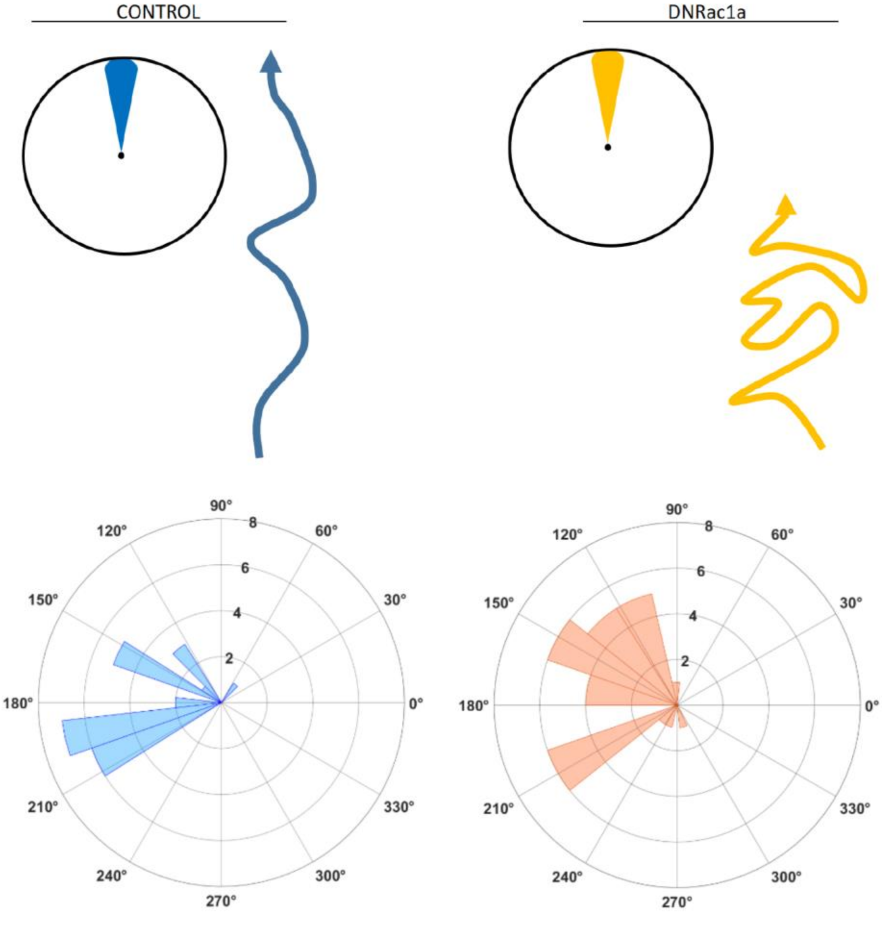

WT polar plot (blue) vs DNRac1a polar plot (orange)

7. *"We extended the analysis by abrogating (DN) and increasing (CA) the function of the*

© 2023. Published by The Company of Biologists under the terms of the Creative Commons Attribution License (https://creativecommons.org/licenses/by/4.0/).

RhoGTPases Cdc42 and Rho1 in otic NB (Fig. 3B-C, Movie 8). In these conditions, we found that NB have a reduced or recovered migratory capacities, respectively (Fig. 3C, asterisk)". There is no quantification of the data for the conditions in which Cdc42 and Rho1 are abrogated or increased. Also, it is unclear what the authors mean by "recovered migratory capacity – does caCdc42 or caRho1 not impair migration or increase motility?

To answer this question, we have included the quantifications regarding the legend of Fig.3B-C: "With respect to the Cdc42 and Rho1 experiments, control cells of the DN condition migrate normal in 2/2 cases; DNCdc42 cells migrate normal in 4/7; DNRho cells migrate normal in 1/4 cases. Control cells of the CA condition migrate normal in 5/5 cases; CACdc42 cells migrate normal in 3/6; CARho cells migrate normal in 1/4 cases" as shown below as **Sup. Fig. 7**.

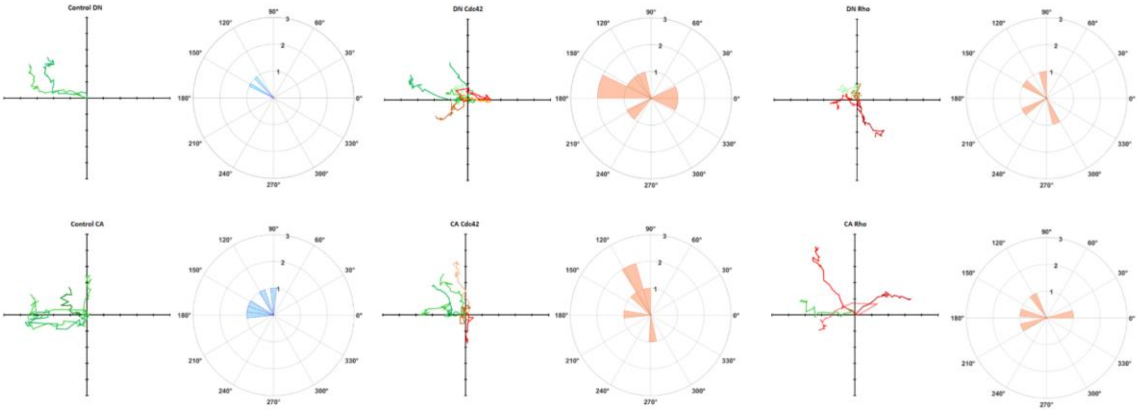

Supplementary figure 7. NB migratory tracks and wind rose plots of directionality in (from left to right and from top to bottom) control, DN Cdc42, DN Rho, control, CA Cdc42 and CA Rho conditions. Adequate migration in migratory tracks is shown in green, while mild and strongly aberrant migration is shown in orange and red, respectively. Control wind rose plots are shown in blue, while altered RhoGTPases wind rose plots are shown in orange.

8- If RhoGTPases regulate the migration of delaminated neuroblasts, what are the consequences of deregulating RhoGTPase for the final formation of SAG?
Our study focuses on single labeled NB migration expressing UAS-RhoGTPases plasmid transiently after injection. Injections of this type generate a mosaic labelling that allow us to analyze individual tracks. To label the whole tissue, we would require a stable transgenic line.
If the effect of lack of RhoGTPases was extensive to the rest of the tissue, we would expect a misshaped SAG with NB migrating aberrantly, which could ultimately affect connectivity to the corresponding hair cell domains. However, it could also be possible that these cells rely, partially or completely, on other robust and redundant mechanisms to generate a proper SAG. We have seen in some cases that when downregulating Rac1a, cells extend more filopodia, which could be due to Cdc42 rescuing the lack of Rac1a in these cells. To what extent lack of RhoGTPases affects later SAG development in terms of final shape is beyond the scope of our study.
We have also thoroughly addressed all grammar and phrasing, as suggested by both reviewer1 and reviewer2. We have also changed the title, as suggested by reviewer2.

Reviewer 2.

We have addressed the main suggestion of reviewer 2 to revise text writing. We have also changed the title, as suggested by reviewer2.
We have added a proper reference for the statement "Thus, for a correct circuitry between hair cells and otic neurons, SAG morphogenesis must be tightly coordinated in time and space with otic tissue development." (Bok et al., 2012)

- *"Does it make sense to call "apical" a domain in a mesenchymal cell?"*

We meant that the apical domain also refers to luminal surface and not intrinsic apical cell domain. We have changed apical for luminal.

© 2023. Published by The Company of Biologists under the terms of the Creative Commons Attribution License (https://creativecommons.org/licenses/by/4.0/).

- The sentence*"unpublished results from the laboratory"* has been removed

-The kymographs in Fig.1H' and H'' are difficult to understand.

Kymograph explanation has been clarified in Materials and Methods section, adding the following: Onto 4D recordings (x, y, z, t) we drawn a line of 1 to 15 pixel thick in our region of interest, and followingly used the Kymograph option in FIJI to deploy a spatial section (X axis) of an image with temporal resolution (Y axis). Data might be interpreted as fluorescent signal crossing the drawn section as we move in time (Y axis). Graphically, time increment in the Y axis equals the spatial thickness of the drawn line.

-"Comparing quantified velocities across time (Fig. 3G left graph and Supplementary figure 4)""across time" can be deleted-
DONE

-I do not understand what they are trying to say in the following paragraph "We have also seen NB can migrate either collectively or non-collectively. In a collective migration, cells can move cryptically within the cluster (Reig et al., 2014) but the overall migration is robust. In NCC tissue, E-cadherin helps maintaining stream migration while Pard3-N-Cadherin interactions avoids crowding by contact-inhibition of locomotion (Clay & Halloran, 2014; Moore et al., 2013; Priya & Yap, 2015; Scarpa et al., 2015). If a temporarily regulated and partial switch in adhesion molecules is necessary to maintain some sort of collective migration for keeping tissue cohesiveness remains to be studied. We do know that itg5a is required for correct aggregation of epibranchial placodes (Bhat & Riley, 2011), maintaining tissue segregation from other placodes and cohesiveness within the tissue."
The paragraph has been modified to this one

In a collective migration, cells in a group move as a coherent entity with some cell reorganizations (Reig et al., 2014). In NCC tissue, E-cadherin helps maintaining stream migration while Pard3-N-Cadherin interactions avoids crowding by contact-inhibition of locomotion (Clay & Halloran, 2014; Moore et al., 2013; Priya & Yap, 2015; Scarpa et al., 2015). In our study, we find that two adjacent migrating NB can detach from neighbours and migrate independently in a non-collective manner, although all cells maintain certain directional coherence. It remains to be explored to what extent cell to cell interactions is required for SAG migration. It is known that itg5a is required for correct aggregation of epibranchial placodes (Bhat & Riley, 2011), maintaining tissue segregation from other placodes and cohesiveness within the tissue, but whether other integrins are also relevant for otic NB cohesiveness and migration is unknown.

"Using in vivo resonant scanning for higher temporal resolution..." This is not correct. Resonant scanning imaging perhaps? In any case, this an other technical details do not need to go in the main text. They seem to have been placed here to create a "wow effect", which in my opinion is not necessary.
Done

Second decision letter

MS ID#: DEVELOP/2023/201824

MS TITLE: Pioneer statoacoustic neurons guide neuroblast behaviour during otic ganglion assembly

AUTHORS: Aitor Bañón and Berta Alsina

I have now received all the referees reports on the above manuscript, and you will see that there are just a couple of issues for you to address prior to publication. The referees' comments are appended below, or you can access them online: please go to BenchPress and click on the

© 2023. Published by The Company of Biologists under the terms of the Creative Commons Attribution License (https://creativecommons.org/licenses/by/4.0/).

'Manuscripts with Decisions' queue in the Author Area. As you will see, one of the comments concerns statistical validity of some of the data; obviously if you have larger numbers for the tracking observations, this issue could be resolved. If not, then please ensure that the descriptions in the results and conclusions made acknowledge any limitations due to relatively low numbers.

Reviewer 1

Advance summary and potential significance to field

see initial submission

Comments for the author

The authors addressed most of the comments and the manuscript is in good shape for publication. a few minor issues are:

-The results in Supplementary Figure. 2 (new data) were not described in the revised manuscript but was only cited. It might be more clear to also discuss these results in the manuscript.

-The sample number for the tracked cells is in many cases quite low (three or four cells). For proper statistics the authors should have an n>10 and also indicate how often the experiment was repeated (many journals ask for N=>3).

Reviewer 2

Advance summary and potential significance to field

No further comments.

Comments for the author

No further comments.

Second revision

Author response to reviewers' comments

Reviewer 1
Comments for the author
The authors addressed most of the comments and the manuscript is in good shape for publication. a few minor issues are:
1) The results in Supplementary Figure. 2 (new data) were not described in the revised manuscript but was only cited. It might be more clear to also discuss these results in the manuscript.
Sup Fig2 description has been included in two sections of results

2) The sample number for the tracked cells is in many cases quite low (three or four cells). For proper statistics the authors should have an n>10 and also indicate how often the experiment was repeated (many journals ask for N=>3).
We have done statistics in Fig3 for DNRac1 overexpression that was the main part of the work (number of cells tracked n= 25 control and n=30 from, 7 and 14 different experimental embryos. With DNRho1 and DnCdc42 we obtained few viable embryos possibly due to vector toxicity, and we did not perform statistics. These results were also treated as supplementary for that reason, complementing in a qualitative manner the results obtained in the main part of the work.

© 2023. Published by The Company of Biologists under the terms of the Creative Commons Attribution License (https://creativecommons.org/licenses/by/4.0/).

In Fig.6 we also have performed statistics on tracked cells, control cells n = 23, n ablated condition cells = 36, from 4 and 10 embryos, from 3 independent experiments.

We would like to point out that these experiments are highly demanding because in addition to overexpressing constructs or ablating cells precisely, it is also required to have good single neuroblasts labelled to be able to track and image overtime. In addition, compared with the CNS, there are much less otic NB that delaminate and migrate, thus numbers of cells to image are much lower.

Third decision letter

MS ID#: DEVELOP/2023/201824

MS TITLE: Pioneer statoacoustic neurons guide neuroblast behaviour during otic ganglion assembly

AUTHORS: Aitor Bañón and Berta Alsina
ARTICLE TYPE: Research Article

I am happy to tell you that your manuscript has been accepted for publication in Development, pending our standard ethics checks.

© 2023. Published by The Company of Biologists under the terms of the Creative Commons Attribution License (https://creativecommons.org/licenses/by/4.0/).
